# A 16S rRNA Gene-Based Metabarcoding of Phosphate-Rich Deposits in Muierilor Cave, South-Western Carpathians

**DOI:** 10.3389/fmicb.2022.877481

**Published:** 2022-05-19

**Authors:** Catalina Haidău, Ruxandra Năstase-Bucur, Paul Bulzu, Erika Levei, Oana Cadar, Ionuţ Cornel Mirea, Luchiana Faur, Victor Fruth, Irina Atkinson, Silviu Constantin, Oana Teodora Moldovan

**Affiliations:** ^1^Department of Biospeleology and Karst Edaphobiology, Emil Racovita Institute of Speleology, Bucureşti, Romania; ^2^Department of Cluj-Napoca, Emil Racovita Institute of Speleology, Cluj-Napoca, Romania; ^3^Romanian Institute of Science and Technology, Cluj-Napoca, Romania; ^4^Department of Molecular Biology and Biotechnology, Faculty of Biology and Geology, Babeş-Bolyai University, Cluj-Napoca, Romania; ^5^Research Institute for Analytical Instrumentation Subsidiary, National Institute of Research and Development for Optoelectronics INOE 2000, Cluj-Napoca, Romania; ^6^Department of Geospeleology and Paleontology, Emil Racovita Institute of Speleology, Bucureşti, Romania; ^7^Faculty of Geology and Geophysics, University of Bucharest, Bucureşti, Romania; ^8^Institute of Physical Chemistry “Ilie Murgulescu” of the Romanian Academy, Bucuresti, Romania; ^9^Centro Nacional Sobre la Evolucion Humana, Burgos, Spain

**Keywords:** cave microbiology, metabarcoding, radiocarbon, Romania, pathogens, fossil bones, bat guano

## Abstract

Muierilor Cave is one of Romania’s most important show caves, with paleontological and archeological deposits. Recently, a new chamber was discovered in the cave, with unique yellow calcite crystals, fine-grained crusts, and black sediments. The deposits in this chamber were related to a leaking process from the upper level that contains fossil bones and a large pile of guano. Samples were taken from the new chamber and another passage to investigate the relationship between the substrate and microbial community. Chemical, mineralogical, and whole community 16S rRNA gene-based metabarcoding analyses were undertaken, and the base of the guano deposit was radiocarbon dated. Our study indicated bacteria linked to the presence of high phosphate concentration, most likely due to the nature of the substrate (hydroxyapatite). Bacteria involved in Fe, Mn, or N cycles were also found, as these elements are commonly identified in high concentrations in guano. Since no bat colonies or fossil bones were present in the new chamber, a high concentration of these elements could be sourced by organic deposits inside the cave (guano and fossil bones) even after hundreds of years of their deposition and in areas far from both deposits. Metabarcoding of the analyzed samples found that ∼0.7% of the identified bacteria are unknown to science, and ∼47% were not previously reported in caves or guano. Moreover, most of the identified human-related bacteria were not reported in caves or guano before, and some are known for their pathogenic potential. Therefore, continuous monitoring of air and floor microbiology should be considered in show caves with organic deposits containing bacteria that can threaten human health. The high number of unidentified taxa in a small sector of Muierilor Cave indicates the limited knowledge of the bacterial diversity in caves that can have potential applications in human health and biotechnology.

## Introduction

Caves are oligotrophic subterranean environments with original biological communities consisting of species with various morphological (i.e., pigmentation and sight loss, body and appendage elongation, etc.), behavioral (i.e., loss of circadian rhythm), and physiological adaptations (i.e., low metabolism rate and tolerance to high CO_2_/low O_2_) (Hershey and Barton, 2018; Howarth and Moldovan, 2018; Friedrich, 2019; Hervant and Malard, 2019; Kowalko, 2019; Zhu et al., 2019). Nowadays, interest in caves has grown proportionally with the curiosity of the people, which is reflected in the growing number of visitors worldwide for the beauty of the subterranean domain and the valuable information it can provide about the past (Gascoyne, 1992; Baldini, 2010; Polyak and Denniston, 2019). Some caves are regarded as great attractions due to their unique morphology (Hill and Forti, 1995; White, 2019; Prelovšek et al., 2021), cultural value (Leroi-Gourhan, 1982; Ronquillo, 1995; Saiz-Jimenez et al., 2011; Skeates and Bergsvik, 2012), or biological diversity (Howarth and Moldovan, 2018; Culver and Pipan, 2019).

The opening of the first show cave in the early 17th century (Vilenica Cave, Slovenia) was just the beginning of the development of speleo-tourism (Cigna, 2019). Since then, more than 600 caves have been visited by tourists worldwide, receiving more than 25 million visitors per year (Cigna and Burri, 2000; Novas et al., 2017; Tièar et al., 2018). With the growing number of tourists, proper management of the caves’ natural heritage is required against the impacts affecting cave habitats and microclimate (Pulido-Bosch et al., 1997; Hoyos et al., 1998; Northup and Lavoie, 2001; Mann et al., 2002; Paksuz and Özkan, 2012; Bercea et al., 2018, 2019; Debata, 2020; Constantin et al., 2021). However, the biological resources of caves can be reservoirs for pathogens and may be regarded as potential environments for pathogen transmission (Dean, 1957; Jurado et al., 2010; Igreja, 2011). Consequently, visitors must be informed of the threats, particularly where the management of show caves is rudimentary (Constantin et al., 2021).

The increasing interest in caves also encompasses the potential biotechnological applications in biological and medical sciences of the cave microbiomes (Jiang et al., 2015; Rangseekaew and Pathom-Aree, 2019; Zada et al., 2021). Already known bacteria reveal new characteristics, and the potential of pathogenicity has been found in some bacteria (Jurado et al., 2010; Moldovan et al., 2020). Alongside these discoveries, unreached sides of metabolism in organisms are starting to be discovered and understood.

Herein, we present the results of the studies on the microbiome of different deposit types in a protected sector of Muierilor Cave (Romania) to unravel the diversity of microorganisms related to the substrate chemistry and mineralogy and identify their possible sources. Muierilor Cave is one of the most important caves from paleontological, biological, and archeological points of view (Mirea et al., 2021). With more than 130,000 visitors each year, it is also one of the most visited show caves in the country (Burghele et al., 2018; Constantin et al., 2021; Mirea et al., 2021).

## Materials and Methods

### Site Description and Sampling

**FIGURE 1:**
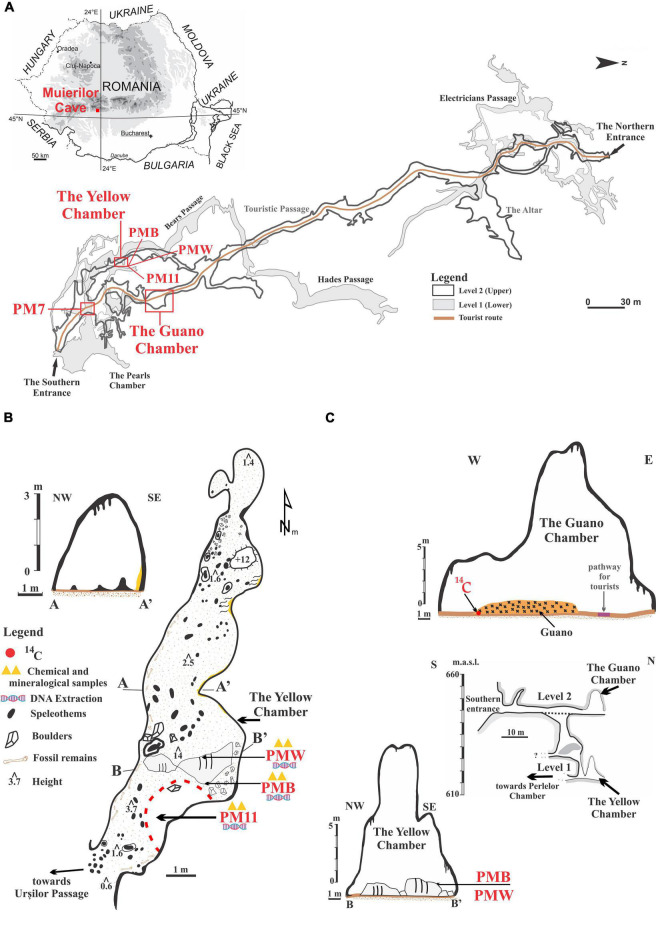
Location of the sampling sites in Muierilor Cave and the country’s position in south-eastern Europe (**A**, cave map courtesy of Grigore Stelian); **(B)** The Yellow Chamber with the sampling locations; **(C)** The Guano Chamber profile, with the position for the ^14^C sample, and the southern profile of the cave showing the overlapping of the Guano Chamber and the Yellow Chamber profile with the sampling locations.

Muierilor Cave (45°11′31.78′′N and 23°45′14.07′′E) is developed in limestone and located in Baia de Fier, south-western Romania, and is one of the most-visited show caves in the country (Figure 1). The cave is situated on the right slope of Galbenul Gorges at 650 m.a.s.l and is developed on four distinct levels. The second one has been a tourist and scientific attraction since the late 19th century. It is the first cave to be fitted with electric lights from Romania since 1963. The cave is inhabited by four species of hibernating bats, with the most abundant being *Miniopterus schreibersii* and *Rhinolophus ferrumequinum*, each forming colonies of around 1,000 individuals, especially along the touristic path.

Muierilor Cave is also known for the rich presence of hydroxyapatite [Ca_5_(PO_4_)_3_(OH)] on rocks and boulders in different sectors of the cave. Hydroxyapatite is a naturally occurring mineral found in the bones and teeth of mammals, which is white when pure and yellow, green, or brown in nature. The lower level is the Scientific Reserve, from where the studied samples were collected, specifically from the Yellow Chamber.

The Yellow Chamber was resurveyed in 2019 during the paleontological excavation in the Scientific Reserve (Mirea et al., 2021). It was named after the yellow calcite crystals that cover the rock surface in some of its parts. The Yellow Chamber is situated roughly underneath the Guano Chamber (which owes its name to a big pile of guano) located along the Touristic Passage (Figure 1). The Touristic Passage is also known for the abundance of fossil remains (belonging mainly to *Ursus spelaeus* sensu lato) (Mirea et al., 2021). Part of the Yellow Chamber floor is covered with a black deposit of sandy clay with very sparse drips of water coming from the upper level, while two big boulders in its middle are covered with white and black crusts at a few tens of centimeters apart. The fragile crusts cover a small surface of the boulders, and at present, no water is dripping in that area. None of the other chambers or passages of the cave have such features, and they have not been seen in other Romanian caves. The ceiling of the Yellow Chamber is about 14 m at the highest point. The walls are covered with seemingly an organic deposit brought by the percolating water from the upper level.

The Yellow Chamber is a side passage with little ventilation. The geomorphological features develop on secondary faults (perpendicular to the main N–S fault) and connect with the upper levels through shafts and fissures. This feature allows the percolating water to precipitate in the lower levels. The temperature at the entrance of the Yellow Chamber (registered with a permanent data logger) was about 10°C, and the humidity was >92%. Spot measurements of CO_2_ were around 560 ppm (Constantin et al., 2021).

Sediment samples were collected in sterile Falcon tubes for microbiological analysis. A sample of 10 g of sediments was taken directly from the Yellow Chamber’s black, sandy clay floor in a Falcon tube (PM11), and about 2 g of each crust was sampled with a sterilized scalpel (PMW = white crust, PMB = black crust). An additional sample of 10 g of brown sandy clay sediments was taken directly in a sterile Falcon tube from a different passage of the Scientific Reserve (PM7). The samples were transported for further laboratory analysis in an icebox and kept in the freezer at –60°C until extraction.

For the chemical and mineralogical analysis, 10 g of sediments from PM7 and PM11 and 2 g of both crusts were collected in clean plastic bags and transported to the laboratory for analysis.

### Dating of Guano Deposits

A guano sample was collected with a small corer from the bottom of the guano pile in the touristic passage for radiocarbon dating. The AMS ^14^C dating was done commercially at the Poznan Radiocarbon Laboratory (Poland), following the procedures described in Mirea et al. (2021). The calibration of AMS ^14^C ages was performed using the program OxCal ver. 4.4.2 (Bronk Ramsey, 2009, 2020) against the INTCAL13 radiocarbon calibration curve (Reimer et al., 2020).

### Chemical and Mineralogical Analysis

The pH was measured using a Seven Excellence Multiparameter (Mettler Toledo, Greifensee, Switzerland) in a 1/5 (m/v) solid to water suspension. N and C were measured using a Flash 2000 CHNS/O analyzer (Thermo Fisher Scientific, Waltham, MA, United States) by combustion of 2 mg of sample. For metal, S, and P analysis, 1 g of dried and grounded sample (<250 μm) was digested in 28 ml of 3/1 (v/v) HCl (37%)/HNO_3_ (62%) mixture. The concentration of Na, Mg, K, Ca, Al, Fe, P, and S was measured by inductively coupled plasma optical emission spectrometry using a 5300 Optima DV (Perkin Elmer, Waltham, MA, United States) spectrometer. The concentration of trace elements was determined by inductively coupled plasma mass spectrometry (ICP-MS) using an Elan DRC II (Perkin Elmer, Waltham, MA, United States) spectrometer.

To preserve crust deposits as well as possible, the mineralogical analysis was performed only on sediment samples. Sampling for mineralogical analysis could lead to the destruction of crusts and subsequently could limit the possibility of further studies. Samples were analyzed using powdered X-ray diffraction method with Rigaku Ultima IV diffractometer in parallel beam geometry equipped with CuKα radiation (wavelength 1.5406 Å). The XRD patterns were collected in the 2θ range between 5 and 80 with a speed of 2°/min and a step size of 0.02°. Rigaku PDXL Software connected to the ICDD database was used for phase identification. The quantitative determination was made using the RIR (reference intensity ratio) methodology.

### DNA Extraction and Sequencing

DNA from sediments (PM7 and PM11) and crusts (PMW and PMB) were extracted according to the following protocol. Sediments were extracted in duplicates, while crusts were extracted in triplicates.

Cells were disrupted using FastPrep-24TM (MP Biomedicals), and genomic DNA was extracted in duplicates from each sample using the commercial kit Quick-DNA Fecal/Soil Miniprep kit (Zymo Research, Irvine, CA, United States), according to the manufacturer’s instructions. DNA was quantified using SpectraMax QuickDrop (Molecular Devices, San Jose, CA, United States) and was further used as a template to investigate the composition of microbial communities in the samples. MiSeq 16S V3–V4 Metagenome Sequencing was performed by a commercial company (Macrogen, Amsterdam, Netherlands). PCR of the V3–V4 hypervariable regions of the bacterial and archeal SSU rRNA gene was performed using primers 341F (50-CCTACGGGNGGCWGCAG-3) and 805R (50-GACTACHVGGGTATCTAATCC-30), according to Illumina’s 16S amplicon-based metagenomics sequencing protocol.

### Metabarcoding Analysis

Sequencing primers from both forward and reverse reads and reads containing any N characters were removed using Cutadapt v2.9 (Martin, 2011). Only reads with a minimum length of 250 nt and a maximum of 301 nt were kept for further analysis. Paired-end reads for the 10 samples were processed using the DADA2 package (Callahan et al., 2016a) implemented in R by adapting existing pipelines [Callahan et al., 2016b; DADA2 Pipeline Tutorial (1.16)]. DADA2 allows accurate differentiation between sequencing errors and true biological variation, thus avoiding the use of operational taxonomic units (OTUs). Instead, DADA2 infers amplicon sequence variants (ASVs). Following primer removal, paired reads were loaded into the DADA2 pipeline and trimmed (forward reads 3′ truncated at 280 nt, reverse reads 3′ truncated at 250 nt), filtered (max. 2 errors per read, minimum length after trimming = 200 nt), and finally merged with a minimum required overlap of 50 nt. Chimeras were removed from merged pairs (1,576 bimeras out of 7,439; 95.8% pass rate). Following filtration and chimera removal, a total of 413,539 merged reads were retained (min per sample = 29,706, max per sample = 55,460, average = 41,353). Taxonomic classification of curated ASVs was performed using the SILVA 138.1 database (Pruesse et al., 2007). From the obtained ASVs, a mean value for triplicates (crusts) and duplicates (sediments) was used in the further analysis. Sequence data generated during this study are available in the European Nucleotide Archive (ENA) as part of project PRJEB51350 with accession numbers ERR9118894–ERR9118903.

### Statistical Analysis

Differences in community composition and statistics were computed using the phyloseq (McMurdie and Holmes, 2013) package in R. Bray distance-based hierarchical clustering and alpha-diversity were estimated after merging replicates by calculating their mean values. Counts and relative abundances were further generated at the genus, class, family, and phylum levels after taxonomic agglomeration using the tax_glom function provided by phyloseq (McMurdie and Holmes, 2013). Taxa merged at each taxonomic rank were considered for abundance estimations only if they were assigned a minimum of 10 counts in at least one averaged sample. Domain *Bacteria* were considered for further analysis of microbial composition. Shannon and Simpson’s diversity indices provided information about the composition of samples by considering both the number of species and the abundance of each species. Shannon’s index gives a better description of a sample’s diversity by considering the species richness and rare species, while Simpson’s index considers evenness and common species. The Chao1 index estimates diversity from abundance data and gives more weight to low-abundance species.

Venn analysis was performed using Web software developed by Heberle et al. (2015). It was used to indicate the distribution of the genera abundances between the different samples. TBtools were used for heatmap representations (Chen et al., 2020).

We used multidimensional scaling (MDS) to represent the sediment samples in a bi-dimensional space. The MDS was done from a similarity matrix between the chemical characteristics of the samples to the coordinates of these in bi-dimensional space for easy visualization. The analysis was done in XLSTAT 2021.4.1 (Addinsoft, New York, NY, United States).

## Results

### Chemical and Mineralogical Analysis

The chemical composition of the samples is presented in Table 1. The pH of both sediments and crusts was slightly alkaline, ranging from 8.3 to 7.4. The sediment sample collected in the Scientific Reserve (PM7) has a slightly higher pH (8.3) than the sediments collected in the Yellow Chamber (PM11, pH = 7.4) and the crusts (pH = 7.7). The carbon content was low (<3%) in the PM11 sediment and the crusts and much higher in PM7 sediment (11.6%), while the N content was low in all the samples (<1%).

**TABLE 1:** Chemical composition of sediment (PM7 and PM11) and crust (PMW and PMB) samples in Muierilor Cave.

Elements	PM7	PM11	PMW	PMB
C (%)	11.6	2	2.84	2.73
N (%)	<0.01	0.84	0.45	0.38
S (mg/kg)	64.7	248	2997	799
Na (mg/kg)	67	397	2485	535
Mg (mg/kg)	282	1208	2275	859
K (mg/kg)	96	3390	2816	678
Ca (mg/kg)	303673	358330	360033	342288
Al (mg/kg)	2537	25183	11931	2758
Fe (mg/kg)	152	25246	8554	739
P (mg/kg)	243	22053	60656	60992
Ba (mg/kg)	44.4	363	111	66.9
Li (mg/kg)	0.96	11.9	9.84	1.87
V (mg/kg)	6.7	24.0	59.7	3.3
Cr (mg/kg)	1.0	24.7	37.7	8.1
Mn (mg/kg)	7.2	240	673	711
Co (mg/kg)	1.0	3.4	10.3	1.7
Ni (mg/kg)	17.9	10.9	93	19.5
Cu (mg/kg)	1.7	286	329	195
Zn (mg/kg)	6.1	559	2251	2387
Ga (mg/kg)	0.06	8.11	3.28	0.7
As (mg/kg)	14.3	5.5	75.3	5.6
Rb (mg/kg)	0.72	37.9	13.1	1.67
Sr (mg/kg)	49.9	138	148	171
Zr (mg/kg)	0.60	7.95	6.56	1.43
Cd (mg/kg)	0.10	2.1	4.4	5.3
Pb (mg/kg)	1.0	11.0	18.9	2.7

The MDS that uses the chemical composition of samples (Supplementary Figure 1) shows the similarity between the crusts separated from the sediment samples. Spatially and thus chemically, PM11 is the nearest to the crusts.

Calcium is the main element present in all the samples, accounting for 30–40% of the sample. The sediment sample collected in the Yellow Chamber (PM11) had higher major and trace element concentrations than the sediment in PM7. Except for Al, Fe, K, and Ba, the sediment in the Yellow Chamber had a lower concentration of metals than the crusts. The high P content in the samples from Yellow Chamber (about 6% in crusts and 2.5% in PM11 sediment) suggests the presence of minerals containing P, most probably hydroxyapatite (Supplementary Figure 2). The PM7 sediment sample has a Ca content similar to that of the crusts, but a much lower P content. The white crust (PMW) has one order of magnitude higher contents of S, Na, Mg, K, Al, and Fe and slightly higher contents of trace elements, suggesting that aluminosilicates constitute these secondary minerals. The high amount of these elements is also reflected in the different abundance of microbial composition in our samples.

X-ray diffraction patterns show that the mineralogical composition of sediments collected from the cave contains mostly primary minerals of detrital origin (Supplementary Figure 2). The mineralogical association is relatively similar, with both samples containing quartz, muscovite, and albite in various concentrations. In addition to the mentioned silicates, the PM11 sample collected from the Yellow Chamber includes hydroxyapatite.

### Guano Deposit Age

The guano sample collected from the bottom of the deposit in the Guano Chamber (Figure 1C) yielded a ^14^C age of 1,315 ± 30 years BP, which corresponds to a calibrated age between AD 654 and 775 (95.4% probability).

### Analysis of Microbial Composition

A total of 5,864 ASVs belonging to *Archaea* (13) and *Bacteria* (5,850) were identified, from which a total of 45 phyla, 105 classes, 221 orders, 265 families, and 464 genera were detected in the four samples. Many ASVs were unclassified at each level, namely 43 phyla (PM7, 16; PM11, 11; PMW, 9; PMB, 7), 332 classes, 901 orders, 2,004 families, and 3,497 genera. Following taxa agglomeration and filtration, 41 phyla, 92 classes, 189 orders, 232 families, and 378 genera were detected in the four averaged samples. Thirty-nine phyla, 90 classes, 187 orders, 231 families, and 378 genera were detected in the four averaged samples.

**FIGURE 2:**
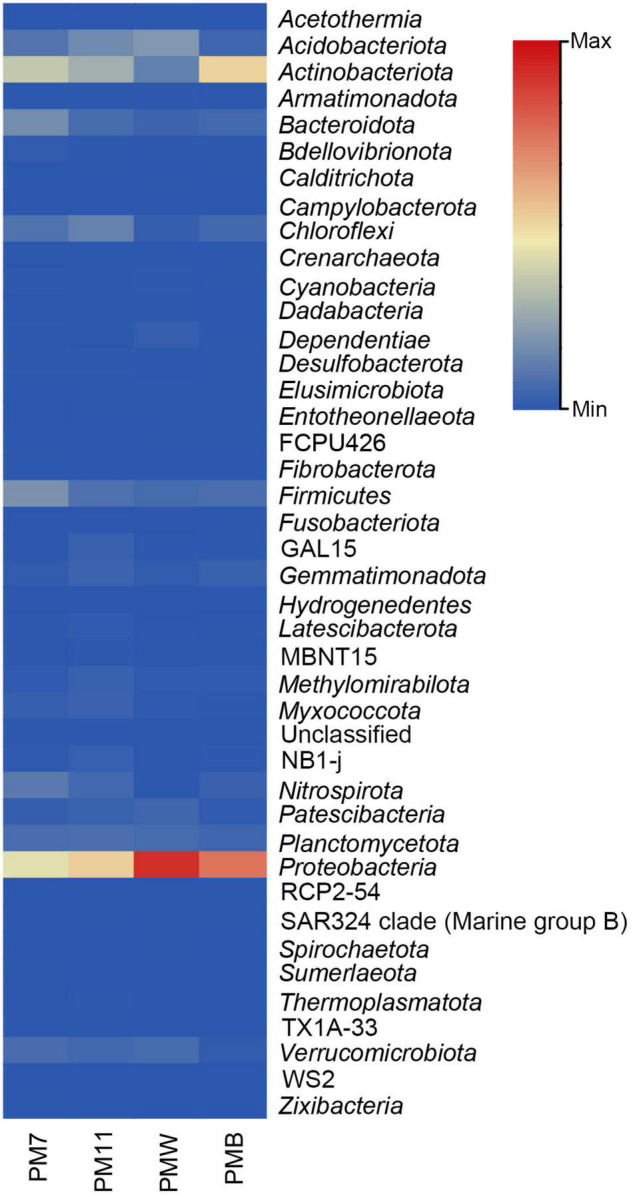
Heatmap of the relative abundance of the different bacterial phyla in the four analyzed samples of Muierilor Cave.

Among all the four samples, *Proteobacteria* was the most abundant phylum (Figure 2), with varied percentages in each sample (PM7, 26%; PM11, 32%; PMW, 55%; and PMB, 45%), followed by *Actinobacteriota* (PM7, 21%; PM11, 17%; PMW, 12%; and PMB, 31%). The third most abundant phyla were *Firmicutes* in PM7 and PMB (11% and 4%) and *Acidobacteriota* in PM11 and PMW (10 and 8%).

Few differences were identified when comparing the presence and absence of phyla, with some present only in sediments or crusts and others present only in one of the samples. *Acetothermia, Campylobacterota*, and *Spirochaetota* were only identified in PMW, while *Fibrobacterota* and *Hydrogenedentes* were observed only in PM7. In addition, *Fusobacteriota* was only found in crust samples, while *Zixibacteria* and TX1A-33 were found in sediment samples.

**FIGURE 3:**
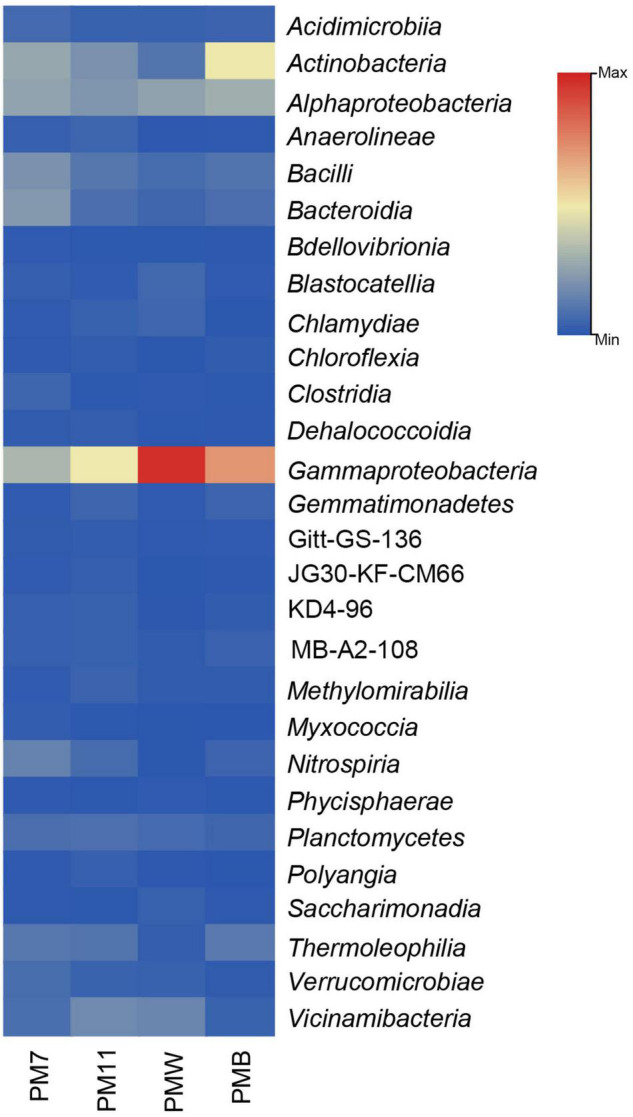
The relative abundance (only > 1% are represented) of the bacterial classes in the four analyzed samples of Muierilor Cave.

At the class level, the relative abundance differed among the samples (Figure 3). In sediment sample PM7, *Gammaproteobacteria* (14%) was the most abundant class, followed by *Actinobacteria* (12%) and *Alphaproteobacteria* (11%). The most abundant class in PM11 was *Gammaproteobacteria* (22%), followed by *Alphaproteobacteria* and *Actinobacteria* (∼9%).

In the crust sample PMW, *Gammaproteobacteria* was the most abundant (∼44%), followed by *Alphaproteobacteria* (12%). In PMB, the most abundant class was also *Gammaproteobacteria* (32%), followed by *Actinobacteria* (22%) and *Alphaproteobacteria* (13%).

**FIGURE 4:**
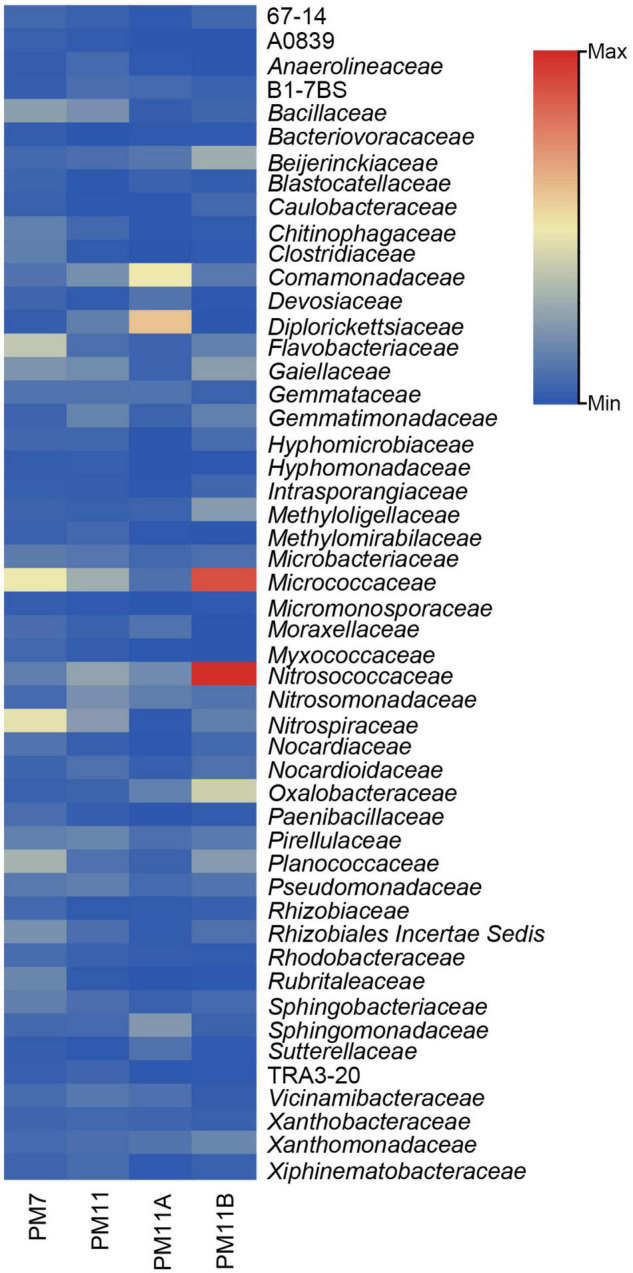
Heatmap of the relative abundance (only > 1% are represented) of bacterial families in the sediment and crust samples of Muierilor Cave.

Families found in high abundance (Figure 4) in the sediment sample PM7 were *Micrococcaceae* and *Nitrospiraceae* (∼7%), *Flavobacteriaceae* and *Planococcaceae* (∼5% and 4%); in PM11 were *Micrococcaceae* (4%), *Nitrosococcaceae* (∼4%), and *Nitrospiraceae* (3%); in the crust sample PMW were *Diplorickettsiaceae* (8%), *Comamonadaceae* (7%), and *Coxiellaceae* (5%); and in PMB were *Nitrosococcaceae* (14%), *Micrococcaceae* (13%), and *Oxalobacteraceae* (6%).

**FIGURE 5:**
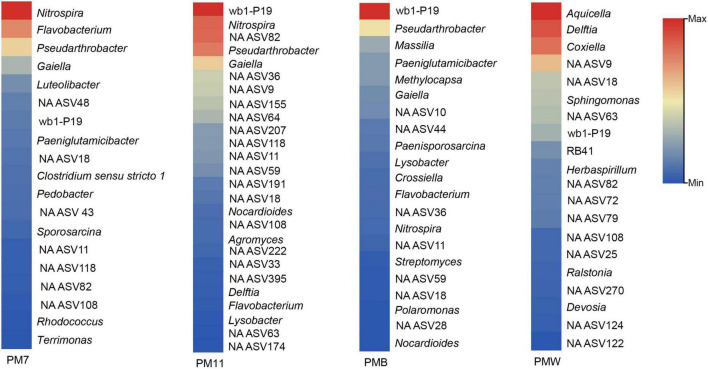
The most abundant genera in each sample of Muierilor Cave (>1%).

The relative abundance of genera was different among the samples and even between the samples of the same type. The most abundant genera in each sample (Figure 5) were *Nitrospira, Flavobacterium*, and *Pseudarthrobacter* in PM7; wb1-P19, *Nitrospira*, NA ASV82, and *Pseudarthrobacter* in PM11; *Aquicella, Delftia*, and *Coxiella* in PMW; and wb1-P19, *Pseudarthrobacter*, and *Massilia* in PMB. The abundance of the total unassigned genera was significantly high (PM7, 31%; PM11, 31%; PMW, 29%; and PMB, 25%), and representatives were found to be among the firsts (Figure 5).

Further analysis indicated the presence of human-related bacteria mainly in Yellow Chamber samples and some presenting potential of being pathogenic (Supplementary Table 1), such as *Actinomyces, Prevotella*, and *Bacteroides*. Almost half of the identified genera were not mentioned to be present in the caves or guano before, with crust samples having the higher abundance (38% in PMW and 25% in PMB).

**FIGURE 6:**
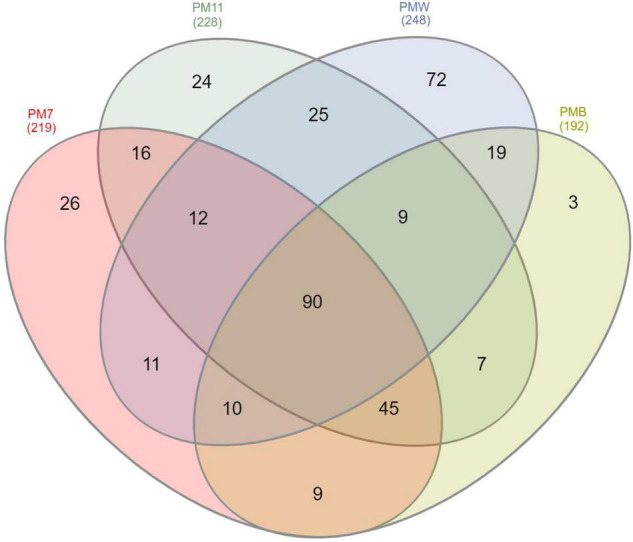
Venn diagram showing the number of shared and unique 16S rRNA-related ASVs in the sediment (PM7 and PM11) and crust (PMW and PMB) samples of Muierilor Cave.

We performed a Venn analysis to better visualize the differences at the genus level (Figure 6). Of the 378 assigned genera, 90 were found in all four samples. The number of genera identified in PM7 (219) was lower than that identified in PM11 (228). The number of genera identified in PMW (248) was higher than in PMB (192). The crust sample PMW (72) had the highest number of unique genera, followed by the sediment samples PM7 (26) and PM11 (24), while PMB (3) had the lowest number.

The calculated alpha diversity indices (Shannon, Chao1, Simpson’s, InvSimpson; Table 2) showed that the bacterial diversity differed between the samples, with a higher diversity in the sediment samples than in the crust samples. Richness estimator, Chao1, showed high values in the sediment samples.

**TABLE 2:** Diversity indices of crust (PMW and PMB) and sediment samples (PM7 and PM11) in Muierilor Cave.

Sample		PMB	PMW	PM11	PM7
Diversity indices	Chao1	1424.0887	1547	2241.0263	1870.0765
	Shannon	5.3614	6.2129	6.8254	6.4270
	Simpson’s	0.9830	0.9935	0.9968	0.9954
	InvSimpson	58.8863	156.2362	320.9038	218.6732

## Discussion

### Chemical and Mineralogical Considerations

The four analyzed samples had a high content of Ca originating from other minerals in this sample, most probably from the limestone rock or calcite dissolution. Among the four samples, only the ones collected from the Yellow Chamber (crusts and PM11) had high P content, which can be attributed to the presence of hydroxyapatite as one of their main constituents. The higher contents of S, Na, Mg, K, Al, Fe, and trace elements in the white crust suggest the presence of aluminosilicates in these secondary minerals. Detrital minerals (quartz, muscovite, and albite) are related to the weathering and transport of soils and geological formations that outcrop outside the cave (Bosch and White, 2004; Kurečić et al., 2021) by running or percolating waters. Hydroxyapatite can be formed in the cave environment by interacting with phosphorus ions and the carbonate component (Audra et al., 2019). The source of phosphate in caves is usually represented either by a fossil bone deposit or a guano accumulation (Hill, 1999; Queffelec et al., 2018). Elements, such as C, P, N, S, K, Na, Cl, Ca, Mg, Fe, Al, Zn, and Ba, were usually identified in guano deposits (Miko et al., 2001; Wurster et al., 2015; Misra et al., 2019), and high concentration of these elements was also found in both the crusts of Muierilor Cave. Considering that no bat colonies or fossil bones are present in the Scientific Reserve, the mechanisms involved in the precipitation of hydroxyapatite in the Yellow Chamber could be related to the presence of organic matter in the above level (the Touristic Passage). The possible source of organic matter in the Touristic Passage is represented by a recent guano deposit (less than c. 1,400 years BP) and fossil bone remains with ages ranging between ∼20,000 and ∼50,000 ka (Mirea et al., 2021). The fossil bone deposits may represent the main source of PO_4_, but the leaching of guano should also be considered. The actual source of phosphate for the Scientific Reserve is challenging to assess, and further studies are required to understand the mechanisms responsible for hydroxyapatite precipitation.

### Microbial Diversity of Sediments vs. Crusts

The microbiological surveys of caves indicate that the most abundant phyla usually found in caves are *Proteobacteria* and *Actinobacteriota* in different proportions, followed by *Firmicutes, Bacteroidota*, and *Chloroflexi* (Porca et al., 2012; Kieraite-Aleksandrova et al., 2015; Oliveira et al., 2017; Dhami et al., 2018; Zhu et al., 2019; Tok et al., 2021), a fact which is consistent with our results. *Proteobacteria* was the most abundant phylum in all the samples and is characterized by a vast metabolic diversity (Kersters et al., 2006; Dong et al., 2020; Tok et al., 2021). *Actinobacteria* was the second most abundant phylum in PM7, PM11, and PMB samples. Members of this phylum are a key community in soil, where they play an important role in the decomposition of organic matter (Madigan et al., 2018; Hazarika and Thakur, 2020; Scheublin et al., 2020). The members of *Acidobacteriota* (second most abundant in PMW and third in PM11) are also known for their involvement in nitrogen assimilation and metabolism of iron, a finding that coincides with the high concentration of N and Fe in our samples (Kielak et al., 2016; Dedysh and Damsté, 2018). The representatives of *Firmicutes* (third most abundant in PM7 and PMB) are commonly found in soils, particularly in the rhizosphere (Kumar et al., 2012). Their presence in caves could be justified by the highly resistant endospores, which are generally unaffected by the environmental stress factors, such as high/low temperatures, unfavorable pH, UV, and lack of water or nutrients (Parkes and Sass, 2009).

Bacterial communities found on the bat’s skin represented the members of the families found in high abundance in our samples, such as members of *Micrococcaceae, Flavobacteriaceae, Comamonadaceae*, and *Planococcaceae* (González-Quiñonez et al., 2014; Lemieux-Labonté et al., 2017). In our samples, families found in high abundance were those previously mentioned in caves or related to bats. For instance, members of *Micrococcaceae* and *Planococcaceae* were sampled from cave walls (Laiz et al., 1999; Armetta et al., 2022), members of *Flavobacteriaceae* from cave water (Shabarova and Pernthaler, 2009), members of *Nitrospiraceae* from speleothem surface (Baskar et al., 2016), and members of *Nitrosococcaceae* from sediment and water (Zhu et al., 2019).

A slight correlation could be drawn when commenting on the relationship between chemistry and microbial composition, and the genera identified mirror the chemical composition of the samples. The Yellow Chamber samples (crusts and PM11) were higher in P, and the identified genera involved in the P cycle (Chen et al., 2006; Sarikhani et al., 2019) showed a higher abundance in these samples than those found in PM7. Such genera are *Chryseobacterium, Enterobacter* (PM11, PMW, and PMB), *Delftia, Stenotrophomonas* (PM11 and PMW), and *Pantoea* (PMW). Higher amounts of Fe, N, and Mn were also found in the samples collected from the Yellow Chamber when compared to the PM7 sediments. Some genera, such as *Rheinheimera* (PMW) and *Ralstonia* (PM11 and PMW), are known to be involved in the iron cycle (Swanner et al., 2011; Schröder et al., 2016), while *Aphanizomenon* NIES81 (PMW), *Herbaspirillum, Methylocella*, and *Ralstonia* (PM11 and PMW) are involved in the N cycle (Ploug et al., 2010; Dalsing et al., 2015; Dedysh and Dunfield, 2016; Waller et al., 2021). Bacteria involved in the Mn cycle (Yang et al., 2013; Bai et al., 2021; Lee et al., 2021) were identified as *Escherichia/Shigella, Halomonas, Microbacterium* (PMW), and *Cupriavidus* (PMW and PMB). Elements, such as N, Fe, and P, are usually found in guano (Miko et al., 2001; Wurster et al., 2015; Misra et al., 2019), P is also associated with bones (Audra et al., 2019), and Mn deposits are usually biogenic in caves (Northup and Lavoie, 2001).

Since we analyzed the chemical differences between PM7 and the Yellow Chamber samples, we could also consider this grouping when discussing human- and bat/guano-related genera. Most bat/guano- and human-related genera were identified in the Yellow Chamber samples (Supplementary Table 1). PM7 was collected from a passage without guano or other organic accumulation.

Bats are a significant reservoir of pathogens and a well-known vector for disease transmission (Ogórek et al., 2018; Agustin et al., 2019). Genera found in crusts, such as *Fusobacterium* and *Rothia*, were previously related to bats, while *Microbacterium* was previously related to guano. Some genera identified in our samples, most of them in crusts, are also found in the guano of other caves in Europe, which are habitats for the same bat species. These are *Enterococcus, Acinetobacter, Pseudomonas, Paenibacillus, Bacillus, Staphylococcus, Rahnella, Micrococcus, Enterobacter, Lysinibacillus*, and *Sphingobacerium* (Tomova et al., 2013; Vandžurová et al., 2013; Veikkolainen et al., 2014; Vengust et al., 2018; Wolkers-Rooijackers et al., 2019; Dimkić et al., 2020; Gerbáčová et al., 2020). Their abundance did not dominate the microbial composition in our samples, but their presence could indicate a possible connection to the upper Guano Chamber.

The presence of a significant number of genera never reported in a cave before (Supplementary Table 1) suggests the limited knowledge regarding the bacterial diversity of caves. Almost half of the genera identified in our samples were never reported in a cave or were never related to bats/guano. Some genera are known members of human microbiota: *Abiotrophia* (Rasic et al., 2020), *Gemella* (García López and Martín-Galiano, 2020), *Granulicatella* (Shailaja et al., 2013), *Treponema* (Seña et al., 2015), and *Haemophilus* (Johnson, 2018). Genera, such as *Bacillus, Escherichia*, and *Staphylococcus*, which are known as “human indicator bacteria,” are usually found in high concentrations after extended cave visits (Lavoie and Northup, 2006; Bercea et al., 2018; Mudgil et al., 2018) and were identified in our samples in high abundance. Recent studies in Muierilor Cave compared the microbial composition in touristic and non-touristic sites. Based on an air survey and human exposed surfaces (Bercea et al., 2018, 2019), the touristic sector of Muierilor Cave fits in the high-risk class (500–2,000 CFU/m^3^, according to the European Commission’s report on Biological Particles in Indoor Environments and Commission of the European Communities, 1994). Moldovan et al. (2020) concluded a clear difference in the microbial composition of water habitats between touristic and non-touristic sites, indicating the strong impact of tourism. These studies found a high abundance of members of *Bacillus* and *Staphylococcus*, which are also abundant in our samples.

Muierilor Cave, as a touristic cave, may be considered as a reservoir of novel and allochthonous species, since animals and humans transit and use it for different purposes. Moreover, visitors play an important role in the spread of bacteria and continuously influence the composition of the microbial community.

## Conclusion

Most of the identified ASVs in four samples, two sediments and two crusts, collected from Muierilor Cave belong to bacteria. The crusts and the nearby sediment sample in a small chamber (the Yellow Chamber) of the cave are phosphate-rich deposits, with abundant bacteria involved in the P cycle. Moreover, bacteria involved in Fe, Mn, and N cycles were found, as these elements are commonly identified in high concentrations in guano. Since no bat colonies or fossil bones were present in the chamber, a high concentration of these elements could be sourced from organic deposits (fossil bones and guano) located at the upper levels of the cave. The high diversity of human-related genera identified only in the crusts and sediment samples collected from the Yellow Chamber, such as *Capnocytophaga, Anaerococcus, Abiotrophia, Actinomyces, Alloprevotella*, and *Eikenella*, could only be related to the upper touristic sector of the cave. Tourism in caves with bat colonies and guano accumulation should benefit from continuous monitoring of the air and floor microbiomes.

Approximately 47% of non-identified bacteria in a small sector of the complex subterranean system of Muierilor Cave indicates the limited knowledge of the bacterial diversity in caves, and these bacteria have high potential in human health and biotechnology applications.

## Data Availability Statement

The datasets presented in this study can be found in online repositories. The names of the repository/repositories and accession number(s) can be found below: ENA – PRJEB51350.

## Author Contributions

OM and CH designed the research. OM, RN-B, CH, IM, and LF collected the field data. RN-B, CH, and PB contributed to the extraction, bioinformatic analysis, and interpretation of the molecular data. EL, OC, LF, VF, and IA contributed to the chemical and mineralogical analyses. SC and IM contributed to the geological context and radiocarbon data interpretation. CH, OM, PB, EL, IM, and LF wrote the first draft. All the authors corrected and approved the final version.

## Conflict of Interest

The authors declare that the research was conducted in the absence of any commercial or financial relationships that could be construed as a potential conflict of interest.

## Publisher’s Note

All claims expressed in this article are solely those of the authors and do not necessarily represent those of their affiliated organizations, or those of the publisher, the editors and the reviewers. Any product that may be evaluated in this article, or claim that may be made by its manufacturer, is not guaranteed or endorsed by the publisher.

## References

[B1] Agustin A. L. D. Atma C. D. Munawaroh M. Ningtyas N. S. Legowo A. P. Sukmanadi M. (2019). Bacterial pathogens from cave-dwelling bats that are a risk to human, animal and environmental health on Lombok Island, Indonesia. *Eur. J. Biosci.* 13 1509–1513.

[B2] Armetta F. Cardenas J. Caponetti E. Alduina R. Presentato A. Vecchioni L. (2022). Conservation state of two paintings in the Santa Margherita cliff cave: role of the environment and of the microbial community. *Environ. Sci. Pollution Res.* 29 29510–29523. 10.1007/s11356-021-17211-0 34751880

[B3] Audra P. De Waele J. Bentaleb I. Chroòáková A. Krištùfek V. D’Angeli I. M. (2019). Guano-related phosphate-rich minerals in European caves. *Int. J. Speleol.* 48 75–105. 10.5038/1827-806X.48.1.2252

[B4] Bai Y. Su J. Wen Q. Huang T. Chang Q. Ali A. (2021). Characterization and mechanism of Mn (II)-based mixotrophic denitrifying bacterium (*Cupriavidus sp*. HY129) in remediation of nitrate (NO3–N) and manganese (Mn (II)) contaminated groundwater. *J. Hazardous Mater.* 408:124414. 10.1016/j.jhazmat.2020.124414 33243652

[B5] Baldini J. U. (2010). The geochemistry of cave calcite deposits as a record of past climate. *Sedimentary Record* 8 4–9. 10.2110/sedred.2010.2.4 30628210

[B6] Baskar S. Routh J. Baskar R. Kumar A. Miettinen H. Itävaara M. (2016). Evidences for microbial precipitation of calcite in speleothems from Krem Syndai in Jaintia Hills, Meghalaya, India. *Geomicrobiol. J.* 33 906–933. 10.1080/01490451.2015.1127447

[B7] Bercea S. Nãstase-Bucur R. Mirea I. C. Mãntoiu D. Ş Kenesz M. Petculescu A. (2018). Novel approach to microbiological air monitoring in show caves. *Aerobiologia* 34 445–468. 10.1007/s10453-018-9523-9

[B8] Bercea S. Nãstase-Bucur R. Moldovan O. T. Kenesz M. Constantin S. (2019). Yearly microbial cycle of human exposed surfaces in show caves. *Subterranean Biol.* 31 1–14. 10.3897/subtbiol.31.34490

[B9] Bosch R. F. White W. B. (2004). “Lithofacies and transport of clastic sediments in karstic aquifers,” in *Studies of Cave Sediments*, eds Sasowsky I. D. Mylroie J. (Boston, MA: Springer), 1–22. 10.1007/978-1-4419-9118-8_1

[B10] Bronk Ramsey C. (2009). Bayesian analysis of radiocarbon dates. *Radiocarbon* 51 337–360. 10.2458/azu_js_rc.51.3494 30854509

[B11] Bronk Ramsey C. (2020). *OxCal Online.* Available online at: https://c14.arch.ox.ac.uk/oxcal.html (Accessed September 7, 2020).

[B12] Burghele B. D. Cucos A. Papp B. Stetca F. A. Mirea I. Constantin S. (2018). Distribution of radon gas in Romanian show caves and radiation safety. *Radiation Protection Dosimetry* 181 1–5. 10.1093/rpd/ncy091 29897577

[B13] Callahan B. J. McMurdie P. J. Rosen M. J. Han A. W. Johnson A. J. A. Holmes S. P. (2016a). DADA2: high-resolution sample inference from Illumina amplicon data. *Nat. Methods* 13 581–583. 10.1038/nmeth.3869 27214047 PMC4927377

[B14] Callahan B. J. Sankaran K. Fukuyama J. A. McMurdie P. J. Holmes S. P. (2016b). Bioconductor workflow for microbiome data analysis: from raw reads to community analyses. *F1000Research* 5:1492. 10.12688/f1000research.8986.2 27508062 PMC4955027

[B15] Chen C. Chen H. Zhang Y. Thomas H. R. Frank M. H. He Y. (2020). TBtools: an integrative toolkit developed for interactive analyses of big biological data. *Mol. Plant* 13 1194–1202. 10.1016/j.molp.2020.06.009 32585190

[B16] Chen Y. P. Rekha P. D. Arun A. B. Shen F. T. Lai W. A. Young C. C. (2006). Phosphate solubilizing bacteria from subtropical soil and their tricalcium phosphate solubilizing abilities. *Appl. Soil Ecol.* 34 33–41. 10.1016/j.apsoil.2005.12.002

[B17] Cigna A. A. (2019). “Chapter 108 - show caves,” in *Encyclopedia of Caves*, 3rd Edn, eds White W. B. Culver D. C. Pipan T. (Waltham, MA: Academic Press), 909–921. 10.1016/B978-0-12-814124-3.00108-4

[B18] Cigna A. A. Burri E. (2000). Development, management and economy of show caves. *Int. J. Speleol.* 29 1–27. 10.5038/1827-806x.29.1.1

[B19] Commission of the European Communities (1994). *Report no. 12: Biological Particles in Indoor Environments.* Luxembourg: Commission of the European Communities.

[B20] Constantin S. Mirea I. C. Petculescu A. Arghir R. A. Mãntoiu D. Ş Kenesz M. (2021). Monitoring human impact in show caves. a study of four Romanian caves. *Sustainability* 13:1619. 10.3390/su13041619

[B21] Culver D. C. Pipan T. (2019). “Survey of subterranean life,” in *The Biology Of Caves And Other Subterranean Habitats*, eds Culver D. C. Pipan T. (Oxford: Oxford University Press), 40–74. 10.1093/oso/9780198820765.003.0003

[B22] *DADA2 Pipeline Tutorial (1.16).* Available online at: http://benjjneb.github.io/dada2/tutorial.html

[B23] Dalsing B. L. Truchon A. N. Gonzalez-Orta E. T. Milling A. S. Allen C. (2015). Ralstonia solanacearum uses inorganic nitrogen metabolism for virulence, ATP production, and detoxification in the oxygen-limited host xylem environment. *mBio* 6:e02471-14. 10.1128/mBio.02471-14 25784703 PMC4453514

[B24] Dean G. (1957). Cave disease. *Central African J. Med.* 3 79–81. 13461069

[B25] Debata S. (2020). Bats in a cave tourism and pilgrimage site in eastern India: conservation challenges. *Oryx* 55 684–691. 10.1017/S003060531900098X

[B26] Dedysh S. N. Damsté J. S. S. (2018). “Acidobacteria,” in *Encyclopedia of Life Sciences*, (Chichester: John Wiley and Sons, Ltd.), 1–10. 10.1002/9780470015902.a0027685

[B27] Dedysh S. N. Dunfield P. F. (2016). “Methylocella,” in *Bergey’s Manual of Systematics of Archaea and Bacteria*, eds Trujillo M. E. Dedysh S. DeVos P. Hedlund B. Kämpfer P. Rainey F. A. (Hoboken, NJ: John Wiley and Sons, Inc), 1–9. 10.1002/9781118960608.gbm00797.pub2

[B28] Dhami N. K. Mukherjee A. Watkin E. L. (2018). Microbial diversity and mineralogical-mechanical properties of calcitic cave speleothems in natural and in vitro biomineralization conditions. *Front. Microbiol.* 9:40. 10.3389/fmicb.2018.00040 29472898 PMC5810276

[B29] Dimkić I. Stanković S. Kabić J. Stupar M. Nenadić M. Ljaljević-Grbić M. (2020). Bat guano-dwelling microbes and antimicrobial properties of the pygidial gland secretion of a troglophilic ground beetle against them. *Appl. Microbiol. Biotechnol.* 104 4109–4126. 10.1007/s00253-020-10498-y 32140841

[B30] Dong Y. Gao J. Wu Q. Ai Y. Huang Y. Wei W. (2020). Co-occurrence pattern and function prediction of bacterial community in Karst cave. *BioMed. Central Microbiol.* 20:137. 10.1186/s12866-020-01806-7 32471344 PMC7257168

[B31] Friedrich M. (2019). “Adaptation to darkness,” in *Encyclopedia of Caves*, 3rd Edn, eds White W. B. Culver D. C. Pipan T. (Cambridge, MA: Academic Press), 16–23. 10.1016/B978-0-12-814124-3.00003-0

[B32] García López E. Martín-Galiano A. J. (2020). The versatility of opportunistic infections caused by Gemella isolates is supported by the carriage of virulence factors from multiple origins. *Front. Microbiol.* 11:524. 10.3389/fmicb.2020.00524 32296407 PMC7136413

[B33] Gascoyne M. (1992). Palaeoclimate determination from cave calcite deposits. *Quaternary Sci. Rev.* 11 609–632. 10.1016/0277-3791(92)90074-I

[B34] Gerbáčová K. Malinièová L. Kisková J. Maslišová V. Uhrin M. Pristaš P. (2020). The faecal microbiome of building-dwelling insectivorous bats (*Myotis myotis* and *Rhinolophus hipposideros*) also contains antibiotic-resistant bacterial representatives. *Curr. Microbiol.* 77 2333–2344. 10.1007/s00284-020-02095-z 32607823

[B35] González-Quiñonez N. Fermin G. Muñoz-Romo M. (2014). Diversity of bacteria in the sexually selected epaulettes of the little yellow-shouldered bat *Sturnira lilium* (Chiroptera: Phyllostomidae). *Interciencia* 39 882–889.

[B36] Hazarika S. N. Thakur D. (2020). “Actinobacteria,” in *Beneficial Microbes in Agro-Ecology*, eds Amaresan N. Senthil Kumar M. Annapurna K. Kumar K. Sankaranarayanan A. (Cambridge, MA: Academic Press), 443–476. 10.1016/B978-0-12-823414-3.00021-6

[B37] Heberle H. Meirelles G. V. da Silva F. R. Telles G. P. Minghim R. (2015). InteractiVenn: a web-based tool for the analysis of sets through Venn diagrams. *BioMed. Central Bioinform.* 16:169. 10.1186/s12859-015-0611-3 25994840 PMC4455604

[B38] Hershey O. S. Barton H. A. (2018). “The microbial diversity of caves,” in *Cave Ecology*, eds Moldovan O. T. Kováè L. Halse S. (Cham: Springer), 69–90. 10.1007/978-3-319-98852-8_5

[B39] Hervant F. Malard F. (2019). “Adaptations: low oxygen,” in *Encyclopedia of Caves*, 3rd Edn, eds White W. B. Culver D. C. Pipan T. (Cambridge, MA: Academic Press), 8–15. 10.1016/B978-0-12-814124-3.00002-9

[B40] Hill A. C. Forti P. (1995). The classification of cave minerals and speleothems. *Int. J. Speleol.* 24 77–82. 10.5038/1827-806x.24.1.5

[B41] Hill C. A. (1999). Mineralogy of Kartchner Caverns, Arizona. *J. Cave Karst Stud.* 61 73–78.

[B42] Howarth G. F. Moldovan T. O. (2018). “The ecological classification of cave animals and their adaptations,” in *Cave Ecology*, eds Moldovan T. O. Kovac L. Halse S. (Berlin: Springer), 41–67. 10.1007/978-3-319-98852-8_4

[B43] Hoyos M. Soler V. Cañaveras J. C. Sánchez-Moral S. Sanz-Rubio E. (1998). Microclimatic characterization of a karstic cave: human impact on microenvironmental parameters of a prehistoric rock art cave (*Candamo Cave*, northern Spain). *Environ. Geol.* 33 231–242. 10.1007/s002540050242

[B44] Igreja R. P. (2011). Infectious diseases associated with caves. *Wilderness Environ. Med.* 22 115–121. 10.1016/j.wem.2011.02.012 21664559

[B45] Jiang Z. K. Guo L. Chen C. Liu S. W. Zhang L. Dai S. J. (2015). Xiakemycin a, a novel pyranonaphthoquinone antibiotic, produced by the *Streptomyces sp*. CC8-201 from the soil of a karst cave. *J. Antibiotics* 68 771–774. 10.1038/ja.2015.70 26104142

[B46] Johnson D. I. (2018). “*Haemophilus spp*,” in *Bacterial Pathogens and Their Virulence Factors*, ed. Johnson D. I. (Cham: Springer), 249–256. 10.1007/978-3-319-67651-7_17

[B47] Jurado V. Laiz L. Rodriguez-Nava V. Boiron P. Hermosin B. Sanchez-Moral S. (2010). Pathogenic and opportunistic microorganisms in caves. *Int. J. Speleol.* 39 15–24. 10.5038/1827-806x.39.1.2

[B48] Kersters K. De Vos P. Gillis M. Swings J. Vandamme P. Stackebrant E. (2006). “Introduction to the proteobacteria,” in *The Prokaryotes*, eds Dworkin M. Falkow S. Rosenberg E. Schleifer K. H. Stackebrandt E. (New York, NY: Springer), 3–37. 10.1007/0-387-30745-1_1

[B49] Kielak A. M. Barreto C. C. Kowalchuk G. A. Van Veen J. A. Kuramae E. E. (2016). The ecology of Acidobacteria: moving beyond genes and genomes. *Front. Microbiol.* 7:744. 10.3389/fmicb.2016.00744 27303369 PMC4885859

[B50] Kieraite-Aleksandrova I. Aleksandrovas V. Kuisiene N. (2015). Down into the Earth: microbial diversity of the deepest cave of the world. *Biologia* 70 989–1002. 10.1515/biolog-2015-0127

[B51] Kowalko E. J. (2019). “Chapter 4 - adaptations: behavioral,” in *Encyclopedia of Caves*, eds White W. Culver D. C. Pipan T. (Waltham, MA: Academic Press), 24–32. 10.1016/B978-0-12-814124-3.00004-2

[B52] Kumar G. Kanaujia N. Bafana A. (2012). Functional and phylogenetic diversity of root-associated bacteria of Ajuga bracteosa in Kangra valley. *Microbiol. Res.* 167 220–225. 10.1016/j.micres.2011.09.001 21968325

[B53] Kurečić T. Bočić N. Wacha L. Bakrač K. Grizelj A. Tresič Pavičić D. (2021). Changes in cave sedimentation mechanisms during the Late Quaternary: an example from the Lower Cerovaèka Cave. Croatia. *Front. Earth Sci.* 9:672229. 10.3389/feart.2021.672229

[B54] Laiz L. Groth I. Gonzalez I. Saiz-Jimenez C. (1999). Microbiological study of the dripping waters in Altamira cave (Santillana del Mar, Spain). *J. Microbiol. Methods* 36 129–138. 10.1016/s0167-7012(99)00018-4 10353807

[B55] Lavoie K. H. Northup D. E. (2006). “Bacteria as indicators of human impact in caves,” in *Proceedings of the 17th National Cave and Karst Management Symposium*, (Albany, NY: The NCKMS Steering Committee), 40–47.

[B56] Lee C. J. Wright M. H. Bentley S. R. Greene A. C. (2021). Draft genome sequence of *Halomonas sp*. strain KAO, a halophilic Mn (II)-oxidizing bacterium. *Microbiol. Resource Announcements* 10:e0032-21. 10.1128/MRA.00032-21 33632853 PMC7909078

[B57] Lemieux-Labonté V. Simard A. Willis C. K. Lapointe J. F. (2017). Enrichment of beneficial bacteria in the skin microbiota of bats persisting with white-nose syndrome. *Microbiome* 5:115. 10.1186/s40168-017-0334-y 28870257 PMC5584028

[B58] Leroi-Gourhan A. (1982). The archaeology of *Lascaux cave*. *Sci. Am.* 246 104–113. 10.1038/scientificamerican0682-104

[B59] Madigan M. T. Bender K. S. Buckley D. H. Sattley W. M. Stahl D. A. (2018). “Chapter 16 – diversity of bacteria,” in *Brock Biology of Microorganisms*, 15th Global Edn, eds Madigan M. T. Bender K. S. Buckley D. H. Sattley W. M. Stahl D. A. (Boston, MA: Benjamin Cummins), 530–565.

[B60] Mann S. L. Steidl R. J. Dalton V. M. (2002). Effects of cave tours on breeding *Myotis velifer*. *J. Wildlife Manag.* 66 618–624. 10.2307/3803128

[B61] Martin M. (2011). Cutadapt removes adapter sequences from high-throughput sequencing reads. *Eur. Mol. Biol. Network J.* 17 10–12. 10.14806/ej.17.1.200

[B62] McMurdie P. J. Holmes S. (2013). phyloseq: an R package for reproducible interactive analysis and graphics of microbiome census data. *Public Library Sci. One* 8:e61217. 10.1371/journal.pone.0061217 23630581 PMC3632530

[B63] Miko S. Kuhta M. Kapelj S. (2001). “Bat guano influence on the geochemistry of cave sediments from Modriè Cave; Croatia,” in *Proceedings of the 13th International Congress of Speleology*, (Brazil).

[B64] Mirea I. C. Robu M. Petculescu A. Kenesz M. Faur L. Arghir R. (2021). Last deglaciation flooding events in the Southern Carpathians as revealed by the study of cave deposits from Muierilor Cave. Romania. *Palaeogeography Palaeoclimatol. Palaeoecol.* 562:110084. 10.1016/j.palaeo.2020.110084

[B65] Misra P. K. Gautam N. K. Elangovan V. (2019). Bat guano: a rich source of macro and microelements essential for plant growth. *Ann. Plant Soil Res.* 21 82–86.

[B66] Moldovan O. T. Bercea S. Nãstase-Bucur R. Constantin S. Kenesz M. Mirea I. C. (2020). Management of water bodies in show caves – a microbial approach. *Tour. Manag.* 78:104037. 10.1016/j.tourman.2019.104037

[B67] Mudgil D. Baskar S. Baskar R. Paul D. Shouche Y. S. (2018). Biomineralization potential of *Bacillus subtilis*, *Rummeliibacillus stabekisii* and *Staphylococcus epidermidis* strains in vitro isolated from speleothems, Khasi Hill Caves, Meghalaya, India. *Geomicrobiol. J.* 35 675–694. 10.1080/01490451.2018.1450461

[B68] Northup D. E. Lavoie K. H. (2001). Geomicrobiology of caves: a review. *Geomicrobiol. J.* 18 199–222. 10.1080/01490450152467750

[B69] Novas N. Gázquez J. A. MacLennan J. García R. M. Fernández-Ros M. Manzano-Agugliaro F. (2017). A real-time underground environment monitoring system for sustainable tourism of caves. *J. Cleaner Product.* 142 2707–2721. 10.1016/j.jclepro.2016.11.005

[B70] Ogórek R. Guz-Regner K. Kokurewicz T. Baraniok E. Kozak B. (2018). Airborne bacteria cultivated from underground hibernation sites in the Nietoperek Bat Reserve (Poland). *J. Cave Karst Stud.* 80 161–171. 10.4311/2017MB0117

[B71] Oliveira C. Gunderman L. Coles C. A. Lochmann J. Parks M. Ballard E. (2017). 16S rRNA gene-based metagenomic analysis of Ozark cave bacteria. *Diversity* 9:31. 10.3390/d9030031 29551950 PMC5856467

[B72] Paksuz S. Özkan B. (2012). The protection of the bat community in the Dupnisa Cave System, Turkey, following opening for tourism. *Oryx* 46 130–136. 10.1017/s0030605310001493

[B73] Parkes R. J. Sass H. (2009). “Deep sub-surface,” in *Encyclopedia of Microbiology*, 3rd Edn, ed. Schaechter M. (Cambridge, MA: Academic Press), 64–79. 10.1007/978-3-642-11274-4_573

[B74] Ploug H. Musat N. Adam B. Moraru L. C. Lavik G. Vagner T. (2010). Carbon and nitrogen fluxes associated with the cyanobacterium *Aphanizomenon sp*. in the Baltic Sea. *Int. Soc. Microbial Ecol.* 4 1215–1223. 10.1038/ismej.2010.53 20428225

[B75] Polyak J. V. Denniston F. R. (2019). “Paleoclimate records from speleothems,” in *Encyclopedia of Caves*, eds White W. Culver D. C. Pipan T. (Waltham, MA: Academic Press), 784–793. 10.1016/B978-0-12-814124-3.00095-9

[B76] Porca E. Jurado V. Žgur-Bertok D. Saiz-Jimenez C. Pašić L. (2012). Comparative analysis of yellow microbial communities growing on the walls of geographically distinct caves indicates a common core of microorganisms involved in their formation. *FEMS Microbiol. Ecol.* 81 255–266. 10.1111/j.1574-6941.2012.01383.x 22486654

[B77] Prelovšek M. Gabrovšek F. Kozel P. Mulec J. Pipan T. Šebela S. (2021). The Škocjan Caves–UNESCO World Heritage Site. *Zeitschrift für Geomorphologie Supplementary Issues* 62 49–64. 10.1127/zfg_suppl/2021/0690

[B78] Pruesse E. Quast C. Knittel K. Fuchs B. M. Ludwig W. Peplies J. (2007). SILVA: a comprehensive online resource for quality checked and aligned ribosomal RNA sequence data compatible with ARB. *Nucleic Acids Res.* 35 7188–7196. 10.1093/nar/gkm864 17947321 PMC2175337

[B79] Pulido-Bosch A. Martin-Rosales W. López-Chicano M. Rodriguez-Navarro C. M. Vallejos A. (1997). Human impact in a tourist karstic cave (Aracena, Spain). *Environ. Geol.* 31 142–149. 10.1007/s002540050173

[B80] Queffelec A. Bertran P. Bos T. Lemée L. (2018). Mineralogical and organic study of bat and chough guano: implications for guano identification in ancient context. *J. Cave Karst Stud. Natl. Speleol. Soc.* 80 49–65. 10.4311/2017ES0102

[B81] Rangseekaew P. Pathom-Aree W. (2019). Cave actinobacteria as producers of bioactive metabolites. *Front. Microbiol.* 10:387. 10.3389/fmicb.2019.00387 30967844 PMC6438885

[B82] Rasic P. Bosnic S. Vasiljevic Z. V. Djuricic S. M. Milickovic M. Savic D. (2020). Abiotrophia defectiva liver abscess in a teenage boy after a supposedly mild blunt abdominal trauma: a case report. *BioMed. Central Gastroenterol.* 20 1–6. 10.1186/s12876-020-01409-6 32795255 PMC7427900

[B83] Reimer P. Austin W. Bard E. Bayliss A. Blackwell P. Bronk Ramsey C. (2020). The IntCal20 Northern Hemisphere radiocarbon age calibration curve (0–55 cal kBP). *Radiocarbon* 62 725–757. 10.1017/RDC.2020.41

[B84] Ronquillo P. W. (1995). Anthropological and cultural values of caves. *Philippine Quarterly Culture Soc.* 23 138–150. 10.1371/journal.pone.0254848 34428206 PMC8384160

[B85] Saiz-Jimenez C. Cuezva S. Jurado V. Fernandez-Cortes A. Porca E. Benavente D. (2011). Paleolithic art in peril: policy and science collide at Altamira Cave. *Science* 334 42–43. 10.1126/science.1206788 21980097

[B86] Sarikhani M. R. Khoshru B. Greiner R. (2019). Isolation and identification of temperature tolerant phosphate solubilizing bacteria as a potential microbial fertilizer. *World J. Microbiol. Biotechnol.* 35:126. 10.1007/s11274-019-2702-1 31363938

[B87] Scheublin T. R. Kielak A. M. van den Berg M. van Veen J. A. de Boer W. (2020). Identification and antimicrobial properties of bacteria isolated from naturally decaying wood. *bioRxiv [preprint]* 10.1101/2020.01.07.896464

[B88] Schröder J. Braun B. Liere K. Szewzyk U. (2016). Draft genome sequence of *Rheinheimera sp*. strain SA_1 isolated from iron backwash sludge in Germany. *Genome Announcements* 4:e00853-16. 10.1128/genomeA.00853-16 27540074 PMC4991719

[B89] Seña A. C. Pillay A. Cox D. L. Radolf J. D. (2015). “*Treponema* and *Brachyspira*, human host-associated *spirochetes*,” in *Manual of Clinical Microbiology*, ed. Jorgensen J. H. Carroll K. C. Funke G. Pfaller M. A. Landry M. L. Richter S. S. (Washington, DC: American Society of Microbiology), 1055–1081. 10.1128/9781555817381.ch60

[B90] Shabarova T. Pernthaler J. (2009). “Investigation of bacterioplankton communities in aquatic karst pools in Bärenschacht cave of Bernese Oberland,” in *Proceedings of the 15th International Congress of Speleology, Kerrville, Texas, July 19-26, 2009*, (Huntsville, AL: National Speleological Society), 416–421.

[B91] Shailaja T. S. Sathiavathy K. A. Unni G. (2013). Infective endocarditis caused by *Granulicatella adiacens*. *Indian Heart J.* 65 447–449. 10.1016/j.ihj.2013.06.014 23993006 PMC3861137

[B92] Skeates R. Bergsvik K. A. (2012). “Chapter 1 caves in context: an introduction,” in *Caves in Context: the Cultural Significance of Caves and Rockshelters in Europe*, eds Skeates S. R. Bergsvik K. A. (Oxford: Oxbow Books), 10.2307/j.ctvh1djk4

[B93] Swanner E. D. Nell R. M. Templeton A. S. (2011). *Ralstonia species* mediate Fe-oxidation in circumneutral, metal-rich subsurface fluids of Henderson mine, CO. *Chem. Geol.* 284 339–350. 10.1016/j.chemgeo.2011.03.015

[B94] Tičar J. Tomič N. Valjavec M. B. Zorn M. Markovič S. B. Gavrilov M. B. (2018). Speleotourism in Slovenia: balancing between mass tourism and geoheritage protection. *Open Geosci.* 10 344–357. 10.1515/geo-2018-0027

[B95] Tok E. Olgun N. Dalfes H. N. (2021). Profiling bacterial diversity in relation to different habitat types in a limestone cave: i̇nsuyu Cave, Turkey. *Geomicrobiol. J.* 38 776–790. 10.1080/01490451.2021.1949647

[B96] Tomova I. Lazarkevich I. Tomova A. Kambourova M. Vasileva-Tonkova E. (2013). Diversity and biosynthetic potential of culturable aerobic heterotrophic bacteria isolated from Magura Cave, Bulgaria. *Int. J. Speleol.* 42 65–76. 10.5038/1827-806X.42.1.8

[B97] Vandžurová A. Baèkor P. Javorskı P. Pristaš P. (2013). Staphylococcus nepalensis in the guano of bats (Mammalia). *Vet. Microbiol.* 164 116–121. 10.1016/j.vetmic.2013.01.043 23462520

[B98] Veikkolainen V. Vesterinen E. J. Lilley T. M. Pulliainen A. T. (2014). Bats as reservoir hosts of human bacterial pathogen, *Bartonella mayotimonensis*. *Emerg. Infect. Dis.* 20 960–967. 10.3201/eid2006.130956 24856523 PMC4036794

[B99] Vengust M. Knapic T. Weese J. S. (2018). The faecal bacterial microbiota of bats; Slovenia. *Public Library Sci. One* 13:e0196728. 10.1371/journal.pone.0196728 29791473 PMC5965822

[B100] Waller S. Wilder S. L. Schueller M. J. Housh A. B. Scott S. Benoit M. (2021). Examining the effects of the nitrogen environment on growth and N2-fixation of endophytic *Herbaspirillum seropedicae* in maize seedlings by applying 11C radiotracing. *Microorganisms* 9:1582. 10.3390/microorganisms9081582 34442661 PMC8401641

[B101] White B. W. (2019). “Speleothems,” in *Encyclopedia of Caves*, eds White W. Culver D. C. Pipan T. (Waltham, MA: Academic Press), 10.1016/B978-0-12-814124-3.00117-5

[B102] Wolkers-Rooijackers J. Rebmann K. Bosch T. Hazeleger W. (2019). Fecal bacterial communities in insectivorous bats from the Netherlands and their role as a possible vector for foodborne diseases. *Acta Chiroptera* 20 475–483. 10.3161/15081109acc2018.20.2.017

[B103] Wurster C. M. Munksgaard N. Zwart C. Bird M. (2015). The biogeochemistry of insectivorous cave guano: a case study from insular Southeast Asia. *Biogeochemistry* 124 163–175. 10.1007/s10533-015-0089-0

[B104] Yang W. Zhang Z. Zhang Z. Chen H. Liu J. Ali M. (2013). Population structure of manganese-oxidizing bacteria in stratified soils and properties of manganese oxide aggregates under manganese–complex medium enrichment. *Public Library Sci. One* 8:e73778. 10.1371/journal.pone.0073778 24069232 PMC3772008

[B105] Zada S. Sajjad W. Rafiq M. Ali S. Hu Z. Wang H. (2021). Cave microbes as a potential source of drugs development in the modern era. *Microb. Ecol.* Online ahead of print. 10.1007/s00248-021-01889-3 34693460 PMC8542507

[B106] Zhu H. Z. Zhang Z. F. Zhou N. Jiang C. Y. Wang B. J. Cai L. (2019). Diversity, distribution and co-occurrence patterns of bacterial communities in a karst cave system. *Front. Microbiol.* 10:1726. 10.3389/fmicb.2019.01726 31447801 PMC6691740

