# Supplementary material for: A 16S rRNA Gene-Based Metabarcoding of Phosphate-Rich Deposits in Muierilor Cave, South-Western Carpathians
Source: Front Microbiol. 2022 May 19;13:877481. doi: 10.3389/fmicb.2022.877481 (PMC9161362; doi:10.3389/fmicb.2022.877481)
Supplement: Supplementary file 1 [file Data_Sheet_1.docx]

***Supplementary Material***

**Figure S1.** The MDS shows the distribution of Muierilor Cave samples based on their chemical composition. The sediment samples (PM7 and PM11) are separated from the crust samples (PMW and PMB). Kruskal's stress (1) = 0.069.


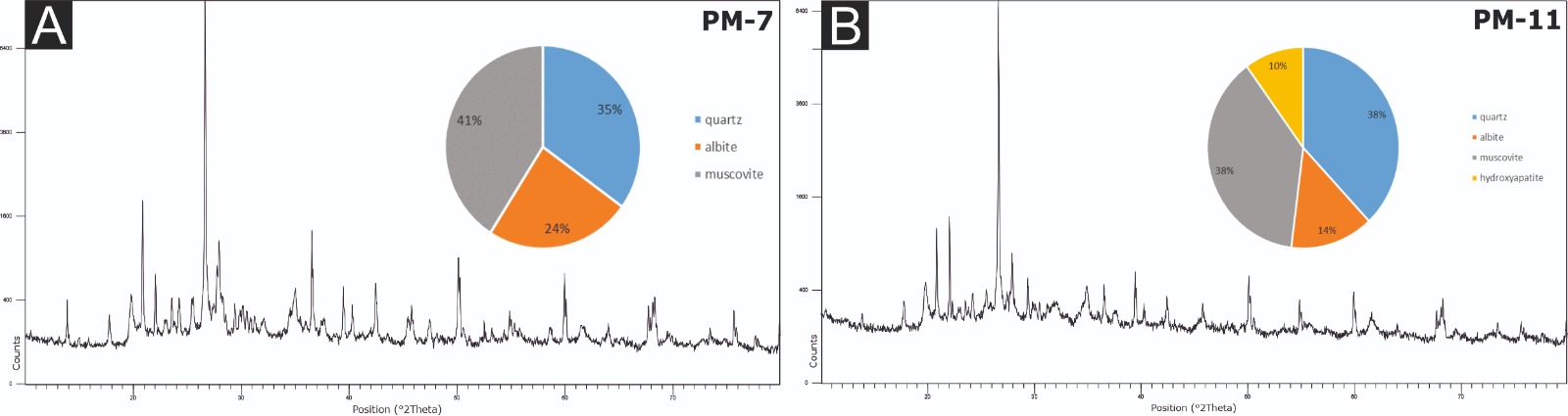


**Figure S2.** XRD results. (A) X-ray diffraction pattern for PM7 sample with derived quantitative analysis shown in the pie chart. (B) X-ray diffraction pattern for PM11 sample with derived quantitative analysis shown in the pie chart.

**Table S1**. Identified bacteria in sediment and crust samples of Muierilor Cave: bold = first mention in a cave, grey = previously mentioned in bats, guano, and caves or are human related; red = pathogenic potential. References for this table are presented below.

| **Genus** | **Samples** | | | | **Human-related** | **Bats** | **Guano** | **Cave** |
| --- | --- | --- | --- | --- | --- | --- | --- | --- |
|  | **PM7** | **PM11** | **PMW** | **PMB** |  |  |  |  |
| ***[Eubacterium] oxidoreducens group*** | |  |  |  |  |  |  |  |
| ***[Ruminococcus] gnavus group*** | |  |  |  |  |  |  |  |
| ***966-1*** |  |  |  |  |  |  |  |  |
| ***Abiotrophia*** |  |  |  |  |  |  |  |  |
| ***Achromobacter*** |  |  |  |  |  |  |  |  |
| ***Acidothermus*** |  |  |  |  |  |  |  |  |
| ***Acidovorax*** |  |  |  |  |  |  |  |  |
| *Acinetobacter* |  |  |  |  |  |  |  |  |
| *Actinomyces* |  |  |  |  |  |  |  |  |
| ***Actinophytocola*** |  |  |  |  |  |  |  |  |
| ***Actinorectispora*** |  |  |  |  |  |  |  |  |
| ***Adhaeribacter*** |  |  |  |  |  |  |  |  |
| ***ADurb.Bin063-1*** |  |  |  |  |  |  |  |  |
| ***Aeromicrobium*** |  |  |  |  |  |  |  |  |
| *Aeromonas* |  |  |  |  |  |  |  |  |
| ***Aetherobacter*** |  |  |  |  |  |  |  |  |
| ***Afipia*** |  |  |  |  |  |  |  |  |
| ***Agromyces*** |  |  |  |  |  |  |  |  |
| ***Ahniella*** |  |  |  |  |  |  |  |  |
| ***AKYG587*** |  |  |  |  |  |  |  |  |
| ***Alkanindiges*** |  |  |  |  |  |  |  |  |
| ***Alloprevotella*** |  |  |  |  |  |  |  |  |
| ***Allorhizobium-Neorhizobium-Pararhizobium-Rhizobium*** |  |  |  |  |  |  |  |  |
| ***Amaricoccus*** |  |  |  |  |  |  |  |  |
| ***Aminobacter*** |  |  |  |  |  |  |  |  |
| ***Amphiplicatus*** |  |  |  |  |  |  |  |  |
| ***Anaerococcus*** |  |  |  |  |  |  |  |  |
| ***Anaerocolumna*** |  |  |  |  |  |  |  |  |
| ***Anaeromyxobacter*** |  |  |  |  |  |  |  |  |
| ***Aphanizomenon NIES81*** | |  |  |  |  |  |  |  |
| ***Aquabacterium*** |  |  |  |  |  |  |  |  |
| ***Aquicella*** |  |  |  |  |  |  |  |  |
| ***Arenimonas*** |  |  |  |  |  |  |  |  |
| ***Arsenicitalea*** |  |  |  |  |  |  |  |  |
| ***Arthrobacter*** |  |  |  |  |  |  |  |  |
| *Asanoa* |  |  |  |  |  |  |  |  |
| ***Atopostipes*** |  |  |  |  |  |  |  |  |
| ***Aurantisolimonas*** |  |  |  |  |  |  |  |  |
| ***Aureibacillus*** |  |  |  |  |  |  |  |  |
| ***Aureimonas*** |  |  |  |  |  |  |  |  |
| ***Bacillus*** |  |  |  |  |  |  |  |  |
| ***Bacteriovorax*** |  |  |  |  |  |  |  |  |
| ***Bacteroides*** |  |  |  |  |  |  |  |  |
| ***Bauldia*** |  |  |  |  |  |  |  |  |
| ***BD1-7 clade*** |  |  |  |  |  |  |  |  |
| ***Bdellovibrio*** |  |  |  |  |  |  |  |  |
| ***Blastocatella*** |  |  |  |  |  |  |  |  |
| ***Blastopirellula*** |  |  |  |  |  |  |  |  |
| ***Bosea*** |  |  |  |  |  |  |  |  |
| ***Bradyrhizobium*** |  |  |  |  |  |  |  |  |
| ***Brevundimonas*** |  |  |  |  |  |  |  |  |
| ***Brochothrix*** |  |  |  |  |  |  |  |  |
| ***Bryobacter*** |  |  |  |  |  |  |  |  |
| ***Buchnera*** |  |  |  |  |  |  |  |  |
| ***Bythopirellula*** |  |  |  |  |  |  |  |  |
| ***Calorithrix*** |  |  |  |  |  |  |  |  |
| ***Campylobacter*** |  |  |  |  |  |  |  |  |
| ***Candidatus Alysiosphaera*** |  |  |  |  |  |  |  |  |
| ***Candidatus Arcanobacter*** | |  |  |  |  |  |  |  |
| ***Candidatus Berkiella*** |  |  |  |  |  |  |  |  |
| ***Candidatus Chloroploca*** | |  |  |  |  |  |  |  |
| ***Candidatus Methylomirabilis*** |  |  |  |  |  |  |  |  |
| ***Candidatus Midichloria*** | |  |  |  |  |  |  |  |
| ***Candidatus Nucleicultrix*** | |  |  |  |  |  |  |  |
| ***Candidatus Omnitrophus*** |  |  |  |  |  |  |  |  |
| ***Candidatus Ovatusbacter*** |  |  |  |  |  |  |  |  |
| ***Candidatus Paracaedibacter*** |  |  |  |  |  |  |  |  |
| ***Candidatus Protochlamydia*** |  |  |  |  |  |  |  |  |
| ***Candidatus Solibacter*** |  |  |  |  |  |  |  |  |
| ***Candidatus Trichorickettsia*** | |  |  |  |  |  |  |  |
| ***Candidatus Xiphinematobacter*** |  |  |  |  |  |  |  |  |
| ***Capnocytophaga*** |  |  |  |  |  |  |  |  |
| ***Catonella*** |  |  |  |  |  |  |  |  |
| ***Caulobacter*** |  |  |  |  |  |  |  |  |
| ***Cavicella*** |  |  |  |  |  |  |  |  |
| ***Cellulomonas*** |  |  |  |  |  |  |  |  |
| ***Cellvibrio*** |  |  |  |  |  |  |  |  |
| ***Cereibacter*** |  |  |  |  |  |  |  |  |
| ***Chryseobacterium*** |  |  |  |  |  |  |  |  |
| ***Chryseolinea*** |  |  |  |  |  |  |  |  |
| ***Chthoniobacter*** |  |  |  |  |  |  |  |  |
| ***Chthonomonas*** |  |  |  |  |  |  |  |  |
| ***Chujaibacter*** |  |  |  |  |  |  |  |  |
| ***CL500-29 marine group*** |  |  |  |  |  |  |  |  |
| ***Cloacibacterium*** |  |  |  |  |  |  |  |  |
| *Clostridium sensu stricto 1* |  |  |  |  |  |  |  |  |
| *Clostridium sensu stricto 13* |  |  |  |  |  |  |  |  |
| *Clostridium sensu stricto 5* |  |  |  |  |  |  |  |  |
| *Clostridium sensu stricto 9* |  |  |  |  |  |  |  |  |
| *Cohnella* |  |  |  |  |  |  |  |  |
| ***Comamonas*** |  |  |  |  |  |  |  |  |
| ***Commensalibacter*** |  |  |  |  |  |  |  |  |
| ***Conexibacter*** |  |  |  |  |  |  |  |  |
| ***Conyzicola*** |  |  |  |  |  |  |  |  |
| ***Corallococcus*** |  |  |  |  |  |  |  |  |
| ***Corynebacterium*** |  |  |  |  |  |  |  |  |
| ***Coxiella*** |  |  |  |  |  |  |  |  |
| ***Crossiella*** |  |  |  |  |  |  |  |  |
| *Cupriavidus* |  |  |  |  |  |  |  |  |
| *Cutibacterium* |  |  |  |  |  |  |  |  |
| ***Deefgea*** |  |  |  |  |  |  |  |  |
| ***Defluviimonas*** |  |  |  |  |  |  |  |  |
| ***Delftia*** |  |  |  |  |  |  |  |  |
| ***Dermacoccus*** |  |  |  |  |  |  |  |  |
| ***DEV114*** |  |  |  |  |  |  |  |  |
| ***Devosia*** |  |  |  |  |  |  |  |  |
| ***Dinghuibacter*** |  |  |  |  |  |  |  |  |
| ***Dokdonella*** |  |  |  |  |  |  |  |  |
| ***Domibacillus*** |  |  |  |  |  |  |  |  |
| ***Dongia*** |  |  |  |  |  |  |  |  |
| ***Duganella*** |  |  |  |  |  |  |  |  |
| ***Dyadobacter*** |  |  |  |  |  |  |  |  |
| ***Edaphobaculum*** |  |  |  |  |  |  |  |  |
| ***Eikenella*** |  |  |  |  |  |  |  |  |
| ***Ellin6055*** |  |  |  |  |  |  |  |  |
| ***Ellin6067*** |  |  |  |  |  |  |  |  |
| ***Enhydrobacter*** |  |  |  |  |  |  |  |  |
| ***Enterobacter*** |  |  |  |  |  |  |  |  |
| *Enterococcus* |  |  |  |  |  |  |  |  |
| ***Epulopiscium*** |  |  |  |  |  |  |  |  |
| ***Erysipelotrichaceae UCG-003*** | |  |  |  |  |  |  |  |
| *Escherichia-Shigella* |  |  |  |  |  |  |  |  |
| ***Faecalibacterium*** |  |  |  |  |  |  |  |  |
| ***Ferruginibacter*** |  |  |  |  |  |  |  |  |
| ***Fimbriiglobus*** |  |  |  |  |  |  |  |  |
| ***Flavihumibacter*** |  |  |  |  |  |  |  |  |
| *Flavobacterium* |  |  |  |  |  |  |  |  |
| ***Fonticella*** |  |  |  |  |  |  |  |  |
| ***Frederiksenia*** |  |  |  |  |  |  |  |  |
| ***Friedmanniella*** |  |  |  |  |  |  |  |  |
| ***Frondihabitans*** |  |  |  |  |  |  |  |  |
| ***Fusobacterium*** |  |  |  |  |  |  |  |  |
| ***Ga0074140*** |  |  |  |  |  |  |  |  |
| *Gaiella* |  |  |  |  |  |  |  |  |
| ***Galbitalea*** |  |  |  |  |  |  |  |  |
| ***Gemella*** |  |  |  |  |  |  |  |  |
| ***Gemmata*** |  |  |  |  |  |  |  |  |
| ***Gemmatimonas*** |  |  |  |  |  |  |  |  |
| ***Gemmobacter*** |  |  |  |  |  |  |  |  |
| ***Glutamicibacter*** |  |  |  |  |  |  |  |  |
| ***Gracilibacillus*** |  |  |  |  |  |  |  |  |
| ***Granulicatella*** |  |  |  |  |  |  |  |  |
| ***Haemophilus*** |  |  |  |  |  |  |  |  |
| ***Haliangium*** |  |  |  |  |  |  |  |  |
| ***Halobacillus*** |  |  |  |  |  |  |  |  |
| ***Haloechinothrix*** |  |  |  |  |  |  |  |  |
| ***Halomonas*** |  |  |  |  |  |  |  |  |
| ***Hassallia*** |  |  |  |  |  |  |  |  |
| *Herbaspirillum* |  |  |  |  |  |  |  |  |
| ***Herbiconiux*** |  |  |  |  |  |  |  |  |
| ***Hirschia*** |  |  |  |  |  |  |  |  |
| ***HSB OF53-F07*** |  |  |  |  |  |  |  |  |
| ***Hydrogenophaga*** |  |  |  |  |  |  |  |  |
| *Hyphomicrobium* |  |  |  |  |  |  |  |  |
| ***Iamia*** |  |  |  |  |  |  |  |  |
| ***Ilumatobacter*** |  |  |  |  |  |  |  |  |
| ***IMCC26207*** |  |  |  |  |  |  |  |  |
| ***Intrasporangium*** |  |  |  |  |  |  |  |  |
| ***IS-44*** |  |  |  |  |  |  |  |  |
| *Janthinobacterium* |  |  |  |  |  |  |  |  |
| ***Jatrophihabitans*** |  |  |  |  |  |  |  |  |
| ***JdFR-76*** |  |  |  |  |  |  |  |  |
| ***Jeongeupia*** |  |  |  |  |  |  |  |  |
| ***JGI 0001001-H03*** |  |  |  |  |  |  |  |  |
| ***Kaistia*** |  |  |  |  |  |  |  |  |
| ***Kineosporia*** |  |  |  |  |  |  |  |  |
| ***Kingella*** |  |  |  |  |  |  |  |  |
| ***Klenkia*** |  |  |  |  |  |  |  |  |
| ***Knoellia*** |  |  |  |  |  |  |  |  |
| *Kocuria* |  |  |  |  |  |  |  |  |
| ***Kribbella*** |  |  |  |  |  |  |  |  |
| ***Lachnoclostridium*** |  |  |  |  |  |  |  |  |
| ***Lacibacter*** |  |  |  |  |  |  |  |  |
| *Lactobacillus* |  |  |  |  |  |  |  |  |
| ***Lacunisphaera*** |  |  |  |  |  |  |  |  |
| ***Lapillicoccus*** |  |  |  |  |  |  |  |  |
| ***Latilactobacillus*** |  |  |  |  |  |  |  |  |
| ***Lawsonella*** |  |  |  |  |  |  |  |  |
| ***LD29*** |  |  |  |  |  |  |  |  |
| ***Legionella*** |  |  |  |  |  |  |  |  |
| ***Leptospirillum*** |  |  |  |  |  |  |  |  |
| *Leptothrix* |  |  |  |  |  |  |  |  |
| ***Leucobacter*** |  |  |  |  |  |  |  |  |
| ***Litorilinea*** |  |  |  |  |  |  |  |  |
| ***Longispora*** |  |  |  |  |  |  |  |  |
| ***Luedemannella*** |  |  |  |  |  |  |  |  |
| ***Luteimonas*** |  |  |  |  |  |  |  |  |
| ***Luteitalea*** |  |  |  |  |  |  |  |  |
| ***Luteococcus*** |  |  |  |  |  |  |  |  |
| *Luteolibacter* |  |  |  |  |  |  |  |  |
| *Lysinibacillus* |  |  |  |  |  |  |  |  |
| ***Lysinimonas*** |  |  |  |  |  |  |  |  |
| *Lysobacter* |  |  |  |  |  |  |  |  |
| ***Marinomonas*** |  |  |  |  |  |  |  |  |
| ***Marmoricola*** |  |  |  |  |  |  |  |  |
| *Massilia* |  |  |  |  |  |  |  |  |
| ***Mesorhizobium*** |  |  |  |  |  |  |  |  |
| ***Methylobacterium-Methylorubrum*** | |  |  |  |  |  |  |  |
| ***Methylocapsa*** |  |  |  |  |  |  |  |  |
| *Methylocella* |  |  |  |  |  |  |  |  |
| ***Methylotenera*** |  |  |  |  |  |  |  |  |
| ***Methylovirgula*** |  |  |  |  |  |  |  |  |
| *Microbacterium* |  |  |  |  |  |  |  |  |
| *Micrococcus* |  |  |  |  |  |  |  |  |
| ***Microlunatus*** |  |  |  |  |  |  |  |  |
| ***Microvirga*** |  |  |  |  |  |  |  |  |
| ***MIZ17*** |  |  |  |  |  |  |  |  |
| ***mle1-7*** |  |  |  |  |  |  |  |  |
| ***MND1*** |  |  |  |  |  |  |  |  |
| ***Mucilaginibacter*** |  |  |  |  |  |  |  |  |
| ***Mycobacterium*** |  |  |  |  |  |  |  |  |
| ***Myxococcus*** |  |  |  |  |  |  |  |  |
| ***Nakamurella*** |  |  |  |  |  |  |  |  |
| ***Nannocystis*** |  |  |  |  |  |  |  |  |
| ***Neisseria*** |  |  |  |  |  |  |  |  |
| ***Neochlamydia*** |  |  |  |  |  |  |  |  |
| *Nitrosomonas* |  |  |  |  |  |  |  |  |
| ***Nitrosospira*** |  |  |  |  |  |  |  |  |
| *Nitrospira* |  |  |  |  |  |  |  |  |
| ***NK4A214 group*** |  |  |  |  |  |  |  |  |
| *Nocardia* |  |  |  |  |  |  |  |  |
| ***Nocardioides*** |  |  |  |  |  |  |  |  |
| ***Nordella*** |  |  |  |  |  |  |  |  |
| ***Noviherbaspirillum*** |  |  |  |  |  |  |  |  |
| ***Novosphingobium*** |  |  |  |  |  |  |  |  |
| ***Oceanicella*** |  |  |  |  |  |  |  |  |
| ***Oerskovia*** |  |  |  |  |  |  |  |  |
| ***Ohtaekwangia*** |  |  |  |  |  |  |  |  |
| ***Oikopleura*** |  |  |  |  |  |  |  |  |
| ***OLB12*** |  |  |  |  |  |  |  |  |
| ***OLB13*** |  |  |  |  |  |  |  |  |
| ***OLB17*** |  |  |  |  |  |  |  |  |
| ***Oligoflexus*** |  |  |  |  |  |  |  |  |
| ***OM27 clade*** |  |  |  |  |  |  |  |  |
| ***OM60(NOR5) clade*** |  |  |  |  |  |  |  |  |
| ***Opitutus*** |  |  |  |  |  |  |  |  |
| ***Oxalicibacterium*** |  |  |  |  |  |  |  |  |
| ***P3OB-42*** |  |  |  |  |  |  |  |  |
| *Paenibacillus* |  |  |  |  |  |  |  |  |
| ***Paeniclostridium*** |  |  |  |  |  |  |  |  |
| ***Paeniglutamicibacter*** |  |  |  |  |  |  |  |  |
| ***Paenisporosarcina*** |  |  |  |  |  |  |  |  |
| ***Pajaroellobacter*** |  |  |  |  |  |  |  |  |
| *Pantoea* |  |  |  |  |  |  |  |  |
| *Paracoccus* |  |  |  |  |  |  |  |  |
| ***Paucibacter*** |  |  |  |  |  |  |  |  |
| ***Pedobacter*** |  |  |  |  |  |  |  |  |
| ***Pedococcus-Phycicoccus*** | |  |  |  |  |  |  |  |
| ***Pedomicrobium*** |  |  |  |  |  |  |  |  |
| ***Pelomonas*** |  |  |  |  |  |  |  |  |
| ***Pelosinus*** |  |  |  |  |  |  |  |  |
| ***Peptoniphilus*** |  |  |  |  |  |  |  |  |
| ***Peptostreptococcus*** |  |  |  |  |  |  |  |  |
| ***Peredibacter*** |  |  |  |  |  |  |  |  |
| ***Phaselicystis*** |  |  |  |  |  |  |  |  |
| ***Phenylobacterium*** |  |  |  |  |  |  |  |  |
| ***Phreatobacter*** |  |  |  |  |  |  |  |  |
| ***Phycisphaera*** |  |  |  |  |  |  |  |  |
| ***Phyllobacterium*** |  |  |  |  |  |  |  |  |
| ***Pir4 lineage*** |  |  |  |  |  |  |  |  |
| ***Pirellula*** |  |  |  |  |  |  |  |  |
| ***Piscinibacter*** |  |  |  |  |  |  |  |  |
| ***Planctomicrobium*** |  |  |  |  |  |  |  |  |
| ***Planctopirus*** |  |  |  |  |  |  |  |  |
| ***Planktothrix NIVA-CYA 15*** | |  |  |  |  |  |  |  |
| ***Planomonospora*** |  |  |  |  |  |  |  |  |
| ***Plasticicumulans*** |  |  |  |  |  |  |  |  |
| ***Pleomorphomonas*** |  |  |  |  |  |  |  |  |
| *Polaromonas* |  |  |  |  |  |  |  |  |
| ***Polycyclovorans*** |  |  |  |  |  |  |  |  |
| ***Porphyromonas*** |  |  |  |  |  |  |  |  |
| *Prevotella* |  |  |  |  |  |  |  |  |
| *Prevotella_7* |  |  |  |  |  |  |  |  |
| ***Pseudaminobacter*** |  |  |  |  |  |  |  |  |
| ***Pseudarthrobacter*** |  |  |  |  |  |  |  |  |
| ***Pseudenhygromyxa*** |  |  |  |  |  |  |  |  |
| ***Pseudoalteromonas*** |  |  |  |  |  |  |  |  |
| *Pseudomonas* |  |  |  |  |  |  |  |  |
| *Pseudonocardia* |  |  |  |  |  |  |  |  |
| ***Pseudorhodobacter*** |  |  |  |  |  |  |  |  |
| ***Pseudorhodoplanes*** |  |  |  |  |  |  |  |  |
| ***Pseudoxanthomonas*** |  |  |  |  |  |  |  |  |
| ***Psychrobacillus*** |  |  |  |  |  |  |  |  |
| ***Psychrobacter*** |  |  |  |  |  |  |  |  |
| ***Psychromonas*** |  |  |  |  |  |  |  |  |
| *Rahnella1* |  |  |  |  |  |  |  |  |
| *Ralstonia* |  |  |  |  |  |  |  |  |
| ***Ramlibacter*** |  |  |  |  |  |  |  |  |
| ***Rathayibacter*** |  |  |  |  |  |  |  |  |
| ***RB41*** |  |  |  |  |  |  |  |  |
| ***Reyranella*** |  |  |  |  |  |  |  |  |
| ***Rheinheimera*** |  |  |  |  |  |  |  |  |
| ***Rhizobacter*** |  |  |  |  |  |  |  |  |
| ***Rhizorhapis*** |  |  |  |  |  |  |  |  |
| ***Rhodobaculum*** |  |  |  |  |  |  |  |  |
| *Rhodococcus* |  |  |  |  |  |  |  |  |
| *Rhodoferax* |  |  |  |  |  |  |  |  |
| ***Rhodomicrobium*** |  |  |  |  |  |  |  |  |
| ***Rhodopirellula*** |  |  |  |  |  |  |  |  |
| ***Rhodoplanes*** |  |  |  |  |  |  |  |  |
| ***Rickettsiella*** |  |  |  |  |  |  |  |  |
| ***Romboutsia*** |  |  |  |  |  |  |  |  |
| ***Roseimicrobium*** |  |  |  |  |  |  |  |  |
| ***Roseobacter clade CHAB-I-5 lineage*** | | |  |  |  |  |  |  |
| ***Roseomonas*** |  |  |  |  |  |  |  |  |
| ***Rothia*** |  |  |  |  |  |  |  |  |
| ***Rubellimicrobium*** |  |  |  |  |  |  |  |  |
| ***Rubrivivax*** |  |  |  |  |  |  |  |  |
| ***Rubrobacter*** |  |  |  |  |  |  |  |  |
| ***Rugamonas*** |  |  |  |  |  |  |  |  |
| ***Salinispora*** |  |  |  |  |  |  |  |  |
| ***Sandaracinus*** |  |  |  |  |  |  |  |  |
| ***Schlesneria*** |  |  |  |  |  |  |  |  |
| ***Serinibacter*** |  |  |  |  |  |  |  |  |
| ***SH3-11*** |  |  |  |  |  |  |  |  |
| ***Shinella*** |  |  |  |  |  |  |  |  |
| ***SH-PL14*** |  |  |  |  |  |  |  |  |
| ***Silvanigrella*** |  |  |  |  |  |  |  |  |
| ***Simplicispira*** |  |  |  |  |  |  |  |  |
| ***Singulisphaera*** |  |  |  |  |  |  |  |  |
| ***SM1A02*** |  |  |  |  |  |  |  |  |
| ***Solibacillus*** |  |  |  |  |  |  |  |  |
| ***Solirubrobacter*** |  |  |  |  |  |  |  |  |
| ***Solitalea*** |  |  |  |  |  |  |  |  |
| ***Sorangium*** |  |  |  |  |  |  |  |  |
| *Sphingobacterium* |  |  |  |  |  |  |  |  |
| ***Sphingobium*** |  |  |  |  |  |  |  |  |
| *Sphingomonas* |  |  |  |  |  |  |  |  |
| ***Sphingopyxis*** |  |  |  |  |  |  |  |  |
| ***Sphingorhabdus*** |  |  |  |  |  |  |  |  |
| ***Sphingosinicella*** |  |  |  |  |  |  |  |  |
| ***Sporichthya*** |  |  |  |  |  |  |  |  |
| ***Sporocytophaga*** |  |  |  |  |  |  |  |  |
| *Sporosarcina* |  |  |  |  |  |  |  |  |
| *Staphylococcus* |  |  |  |  |  |  |  |  |
| ***Stenotrophobacter*** |  |  |  |  |  |  |  |  |
| *Stenotrophomonas* |  |  |  |  |  |  |  |  |
| *Steroidobacter* |  |  |  |  |  |  |  |  |
| *Streptococcus* |  |  |  |  |  |  |  |  |
| *Streptomyces* |  |  |  |  |  |  |  |  |
| ***Subgroup 10*** |  |  |  |  |  |  |  |  |
| *Sulfurifustis* |  |  |  |  |  |  |  |  |
| ***Sumerlaea*** |  |  |  |  |  |  |  |  |
| ***SWB02*** |  |  |  |  |  |  |  |  |
| ***Tabrizicola*** |  |  |  |  |  |  |  |  |
| ***Tepidimonas*** |  |  |  |  |  |  |  |  |
| ***Terrimicrobium*** |  |  |  |  |  |  |  |  |
| ***Terrimonas*** |  |  |  |  |  |  |  |  |
| ***Thermomonas*** |  |  |  |  |  |  |  |  |
| ***Thioalkalimicrobium*** |  |  |  |  |  |  |  |  |
| *Thiobacillus* |  |  |  |  |  |  |  |  |
| ***TM7a*** |  |  |  |  |  |  |  |  |
| ***Tomitella*** |  |  |  |  |  |  |  |  |
| ***Treponema*** |  |  |  |  |  |  |  |  |
| ***Tumebacillus*** |  |  |  |  |  |  |  |  |
| ***Tundrisphaera*** |  |  |  |  |  |  |  |  |
| ***Urania-1B-19 marine sediment group*** | | |  |  |  |  |  |  |
| ***UTBCD1*** |  |  |  |  |  |  |  |  |
| ***UTCFX1*** |  |  |  |  |  |  |  |  |
| *Variovorax* |  |  |  |  |  |  |  |  |
| ***Veillonella*** |  |  |  |  |  |  |  |  |
| ***Verrucomicrobium*** |  |  |  |  |  |  |  |  |
| ***Vibrio*** |  |  |  |  |  |  |  |  |
| *Vicinamibacter* |  |  |  |  |  |  |  |  |
| ***Virgibacillus*** |  |  |  |  |  |  |  |  |
| ***Vulgatibacter*** |  |  |  |  |  |  |  |  |
| ***wb1-A12*** |  |  |  |  |  |  |  |  |
| *wb1-P19* |  |  |  |  |  |  |  |  |
| *Weissella* |  |  |  |  |  |  |  |  |
| ***Woeseia*** |  |  |  |  |  |  |  |  |
| ***Youhaiella*** |  |  |  |  |  |  |  |  |

Alonso, L., Creuzé-des-Châtelliers, C., Trabac, T. *et al.* Rock substrate rather than black stain alterations drives microbial community structure in the passage of Lascaux Cave. Microbiome 6**,**216 (2018). <https://doi.org/10.1186/s40168-018-0599-9>

Banskar, S., Bhute, S., Suryavanshi, M. Punekar, S., Shouche, Y. S. (2016). Microbiome analysis reveals the abundance of bacterial pathogens in *Rousettus leschenaultii* guano. Science Reports, 6. doi: <https://doi.org/10.1038/srep36948>

Barra, W.F., Sarquis, D.P., Khayat, A.S., Khayat, B.C.M., Demachki, S., Anaissi, A.K.M., et al. (2021). Gastric Cancer Microbiome. Pathobiology, 88, 156-169. doi: <https://doi.org/10.1159/000512833>

Barton, H. A. (2006). Introduction to cave microbiology: a review for the non-specialist. Journal of cave and karst studies, 68, 43-54.

Barton, H. A., Taylor, N. M., Kreate, M. P., Springer, A. C., Oehrle, S. A., Bertog, J. L. (2007). The impact of host rock geochemistry on bacterial community structure in oligotrophic cave environments. International Journal of Speleology, 36, 5.doi: <http://dx.doi.org/10.5038/1827-806X.36.2.5>

Baskar, S., Chalia, S., Baskar, R. (2018). Calcite precipitation by *Rhodococcus sp*. isolated from Kotumsar cave, Chhattisgarh, India. Current Science, 1063-1074.

Bastian, F., Jurado, V., Nováková, A., Alabouvette, C., Sáiz-Jiménez, C. (2010). The microbiology of Lascaux cave. Microbiology, 156, 644-652.doi: <https://doi.org/10.1099/mic.0.036160-0>

Becker, K., Skov, R.L. and von Eiff, C., (2015). *Staphylococcus*, *Micrococcus*, and other catalase‐positive cocci, in Manual of clinical microbiology, ed. J.H., Jorgensen, K.C., Carroll, G., Funke, M.A., Pfaller, M.L., Landry, S.S., Richter, D.W., Warnockpp. (11th ed. ASM Press, Washington, DC) 354-382. doi: <https://doi.org/10.1128/9781555817381.ch21>

Benavides JA, Shiva C, Virhuez M, Tello C, Appelgren A, Vendrell J, et al. (2018). Extended-spectrum beta-lactamase-producing *Escherichia coli* in common vampire bats *Desmodus rotundus* and livestock in Peru. Zoonoses Public Health, 654, 454-458. doi: 10.1111/zph.12456

Borda, D.R., Nastase-Bucur, R.M., Spinu, M., Uricariu, R., Mulec, J. (2014). Aerosolized microbes from organic rich materials: Case study of bat guano from caves in Romania. Journal of Cave and Karst Studies, 76.doi: 10.4311/2013MB0116

Brooks, R.S., Blanchard, M.T., Clothier, K.A., Fish, S., Anderson, M.L., Stott, J.L. (2016). Characterization of *Pajaroellobacter abortibovis*, the etiologic agent of epizootic bovine abortion. Veterinary microbiology, 192, 73-80. doi: <https://doi.org/10.1016/j.vetmic.2016.07.001>

Cacchio, P., Ercole, C., Cappuccio, G., Lepidi, A. (2003). Calcium carbonate precipitation by bacterial strains isolated from a limestone cave and from a loamy soil. Geomicrobiology Journal, 20, 85-98. doi: <https://doi.org/10.1080/01490450303883>

Carmichael, M. J., Carmichael, S. K., Santelli, C. M., Strom, A., Bräuer, S. L. (2013). Mn (II)-oxidizing bacteria are abundant and environmentally relevant members of ferromanganese deposits in caves of the upper Tennessee River Basin. Geomicrobiology Journal, 30, 779-800.doi: <https://doi.org/10.1080/01490451.2013.769651>

Chalia, S., Baskar, S., Minakshi, P., Baskar, R., Ranjan, K. (2017). Biomineralization abilities of *Cupriavidus* strain and *Bacillus subtilis* strains in vitro isolated from speleothems, Rani Cave, Chhattisgarh, India. Geomicrobiology Journal, 34, 737-752. doi: <https://doi.org/10.1080/01490451.2016.1257663>

Chen, W., Zhao, Y. L., Cheng, J., Zhou, X. K., Salam, N., Fang, B. Z., et al. (2016). *Lysobacter cavernae sp*. nov., a novel bacterium isolated from a cave sample. Antonie van Leeuwenhoek, 109, 1047-1053.doi: https://doi.org/10.1007/s10482-016-0704-7

Chen, Y., Wu, L., Boden, R., Hillebrand, A., Kumaresan, D., Moussard, H., et al. (2009). Life without light: microbial diversity and evidence of sulfur-and ammonium-based chemolithotrophy in Movile Cave. International Society for Microbial Ecology, 3, 1093-1104.doi: <https://doi.org/10.1038/ismej.2009.57>

Christensen, J.J., Ruoff, K.L. (2015). “*Aerococcus*, *Abiotrophia*, and Other Aerobic Catalase‐Negative, Gram‐Positive Cocci” in Manual of Clinical Microbiology, ed. J.H., Jorgensen, K.C., Carroll, G., Funke, M.A., Pfaller, M.L., Landry, S.S., Richter, D.W., Warnock (11th ed. ASM Press, Washington, DC), 422-436. doi: <https://doi.org/10.1128/9781555817381.ch24>

Cohen-Poradosu, R., Kasper, L.D. (2015). “Anaerobic Infections: General Concepts” in Mandell, Douglas, and Bennett's Principles and Practice of Infectious Diseases, ed. J.E., Bennett, R., Dolin, M.J., Blaser, (Eighth Edition, Elsevier, Philadephia, PA), 2923-2932.

Conville, P.S., Witebsky, F.G. (2015). “*Nocardia, Rhodococcus, Gordonia, Actinomadura, Streptomyces*, and other aerobic actinomycetes” in Manual of clinical microbiology, ed. J.H., Jorgensen, K.C., Carroll, G., Funke, M.A., Pfaller ,M.L., Landry, S.S., Richter, D.W., Warnock (11th ed. ASM Press, Washington, DC), 504-535.

Daniel, D.S., Ng, Y.K., Chua, E.L., Arumugam, Y., Wong, W.L., Kumaran, J.V. (2013). Isolation and identification of gastrointestinal microbiota from the short-nosed fruit bat *Cynopterus brachyotis brachyotis*. Microbiological research, 168, 485-496. doi: <https://doi.org/10.1016/j.micres.2013.04.001>

De Mandal, S., Chatterjee, R., Kumar, N. S. (2017). Dominant bacterial phyla in caves and their predicted functional roles in C and N cycle. BioMed Central Microbiology, 17, 90. doi: <https://doi.org/10.1186/s12866-017-1002-x>

Dimkić, I., Stanković, S., Kabić, J., Stupar, M., Nenadić, M., Ljaljević-Grbić, M., et al. (2020). Bat guano-dwelling microbes and antimicrobial properties of the pygidial gland secretion of a troglophilic ground beetle against them. Applied Microbiology and Biotechnology 104, 4109–4126. <https://doi.org/10.1007/s00253-020-10498-y>

Elias, J., Frosch, M. and Vogel, U. (2015). “*Neisseria*” in Manual of clinical microbiology, ed. J.H. Jorgensen, K.C. Carroll, G. Funke, M.A., Pfaller, M.L., Landry, S.S., Richter, D.W., Warnock (ASM Press, Washington, DC, USA), 635-651. doi: <https://doi.org/10.1128/9781555817381.ch34>

Gan, H. Y., Gan, H. M., Tarasco, A. M., Busairi, N. I., Barton, H. A., Hudson, A. O., Savka, M. A. (2014). Whole-genome sequences of five oligotrophic bacteria isolated from deep within Lechuguilla Cave, New Mexico. Genome announcements, 2(6).doi: <https://doi.org/10.1128/genomeA.01133-14>

Ghosh, S., Paine, E., Wall, R., Kam, G., Lauriente, T., Sa-Ngarmangkang, P. C. et al. (2017). In situ cultured bacterial diversity from iron curtain cave, Chilliwack, British Columbia, Canada. Diversity, 9, 36.doi: <https://doi.org/10.3390/d9030036>

Gutierrez-Patricio, S., Gonzalez-Pimentel, J. L., Miller, A. Z., Hermosin, B., Saiz-Jimenez, C., Jurado, V. (2021). *Paracoccus onubensis* sp. nov., a novel alphaproteobacterium isolated from the wall of a show cave. International journal of systematic and evolutionary microbiology, 71.doi: <https://dx.doi.org/10.1099%2Fijsem.0.004942>

Hall, V. and Copsey, S.D. (2015). “*Propionibacterium, Lactobacillus, Actinomyces*, and Other Non‐Spore‐Forming Anaerobic Gram‐Positive Rods” in Manual of clinical microbiology, ed. J.H. Jorgensen, K.C. Carroll, G. Funke, M.A., Pfaller, M.L., Landry, S.S., Richter, D.W., Warnock (ASM Press, Washington, DC, USA), 920-939. doi: <https://doi.org/10.1128/9781555817381.ch52>

Hamedi, J., Kafshnouchi, M., Ranjbaran, M. (2019). A study on actinobacterial diversity of Hampoeil cave and screening of their biological activities. Saudi journal of biological sciences, 26, 1587-1595. doi: <https://doi.org/10.1016/j.sjbs.2018.10.010>

Jurado, V., Gonzalez-Pimentel, J. L., Miller, A. Z., Hermosin, B., D’Angeli, I. M., Tognini, P., et al. (2020). Microbial communities in vermiculation deposits from an Alpine cave. Frontiers in Earth Science, 635.doi: https://doi.org/10.3389/feart.2020.586248

Jurado, V., Gonzalez-Pimentel, J. L., Miller, A. Z., Hermosin, B., D’Angeli, I. M., Tognini, P., et al. (2020). Microbial communities in vermiculation deposits from an Alpine cave. Frontiers in Earth Science, 635.doi: <https://doi.org/10.3389/feart.2020.586248>

Kondratyeva, L. M., Polevskaya, O. S., Litvinenko, Z. N., Golubeva, E. M., Konovalova, N. S. (2016). Role of the microbial community in formation of speleothem (moonmilk) in the Snezhnaya carst cave (Abkhazia). Microbiology, 85, 629-637.doi: <https://doi.org/10.1134/S002626171605009X>

Könönen, E., Conrads, G., Nagy, E. (2015). “*Bacteroides, Porphyromonas, Prevotella, Fusobacterium*, and Other Anaerobic Gram‐Negative Rods” in Manual of clinical microbiology, ed. J.H. Jorgensen, K.C. Carroll, G. Funke, M.A., Pfaller, M.L., Landry, S.S., Richter, D.W., Warnock (ASM Press, Washington, DC, USA), 967-993. doi: <https://doi.org/10.1128/9781555817381.ch54>

Laiz, L., Groth, I., Gonzalez, I., Saiz-Jimenez, C. (1999). Microbiological study of the dripping waters in Altamira cave (Santillana del Mar, Spain). Journal of microbiological methods, 36, 129-138. doi: <https://doi.org/10.1016/s0167-7012(99)00018-4>

Ledeboer, N.A. and Doern, G.V. (2015). “*Haemophilus*” in Manual of clinical microbiology, ed. J.H. Jorgensen, K.C. Carroll, G. Funke, M.A., Pfaller, M.L., Landry, S.S., Richter, D.W., Warnock (ASM Press, Washington, DC, USA), 667-684. doi: <https://doi.org/10.1128/9781555817381.ch36>

Li, M., Fang, C., Kawasaki, S., Huang, M., Achal, V. (2019). Bio-consolidation of cracks in masonry cement mortars by Acinetobacter sp. SC4 isolated from a karst cave. International Biodeterioration and Biodegradation, 141, 94-100.doi: <https://doi.org/10.1016/j.ibiod.2018.03.008>

Manolache, E., Onac, B. P. (2000). Geomicrobiology of black sediments in Vantului Cave (Romania): preliminary results. Cave and Karst Science, 27, 109-112.

Miller, A. Z., García-Sánchez, A. M., L Coutinho, M., Costa Pereira, M. F., Gázquez, F., Calaforra, J. M., et al. (2020). Colored microbial coatings in show caves from the Galapagos Islands (Ecuador): first microbiological approach. Coatings, 10, 1134.doi: https://doi.org/10.3390/coatings10111134

Mühldorfer, K. (2013). Bats and bacterial pathogens: a review. Zoonoses and public health, 60, 93-103. doi: <https://doi.org/10.1111/j.1863-2378.2012.01536.x>

Murphy E.C., FrickI.M. (2013) Gram-positive anaerobic cocci – commensals and opportunistic pathogens, Federation of European Microbiological Societies, Microbiology Reviews, 37, 520–553. doi: https://doi.org/10.1111/1574-6976.12005

Newman, M.M., Kloepper, L.N., Duncan, M., McInroy, J.A., Kloepper, J.W. (2018). Variation in bat guano bacterial community composition with depth. Frontiers in microbiology, 9. doi: <https://doi.org/10.3389/fmicb.2018.00914>

Northup, D.E., Barns, S.M., Yu, L.E., Spilde, M.N., Schelble, R.T., Dano, K.E., et al. (2003). Diverse microbial communities inhabiting ferromanganese deposits in Lechuguilla and Spider Caves. Environmental Microbiology, 5, 1071-1086. doi: <https://doi.org/10.1046/j.1462-2920.2003.00500.x>

Paul, K., and Patel, S. S. (2001). *Eikenella corrodens* infections in children and adolescents: case reports and review of the literature. Clinical infectious diseases, 33, 54-61. Doi: <https://doi.org/10.1086/320883>

Rahman, M. T., Crombie, A., Chen, Y., Stralis-Pavese, N., Bodrossy, L., Meir, P., et al. (2011). Environmental distribution and abundance of the facultative methanotroph *Methylocella*. International Society for Microbial Ecology, 5, 1061–1066.doi: <https://doi.org/10.1038/ismej.2010.190>

Rao, M. P. N., Dong, Z. Y., Kan, Y., Zhang, K., Fang, B. Z., Xiao, M., et al. (2020). Description of *Paenibacillus antri* sp. nov. and Paenibacillus mesophilus sp. nov., isolated from cave soil. International Journal of Systematic and Evolutionary Microbiology, 70, 1048-1054. doi: <https://doi.org/10.1099/ijsem.0.003870>

Sakoui, S., Derdak, R., Addoum, B., Pop, O. L., Vodnar, D. C., Suharoschi, R., et al. (2022). The first study of probiotic properties and biological activities of lactic acid bacteria isolated from Bat guano from Er-rachidia, Morocco. Food Science and Technology, 159.doi: <https://doi.org/10.1016/j.lwt.2022.113224>

Seña, A.C., Pillay, A., Cox, D.L., Radolf, J.D. (2015). “*Treponema* and *Brachyspira*, Human Host‐Associated *Spirochetes*” in Manual of clinical microbiology, ed. J.H. Jorgensen, K.C. Carroll, G.Funke, M.A. Pfaller, M.L. Landry, S.S. Richter, D.W. Warnock. 1055-1081. Doi: <https://doi.org/10.1128/9781555817381.ch60>

Senn, L., Entenza, J.M., Greub, G., Jaton, K., Wenger, A., Bille, J., Calandra, T., Prod'hom, G. (2006). Bloodstream and endovascular infections due to *Abiotrophia defectiva* and *Granulicatella* species. BioMed Central Infectious Diseases, 6, 1-6. doi: <https://doi.org/10.1186/1471-2334-6-9>

Song, Y., Finegold, S.M. (2015). “*Peptostreptococcus, Finegoldia, Anaerococcus, Peptoniphilus, Veillonella*, and other anaerobic cocci” in Manual of clinical microbiology, ed. J. H. Jorgensen, K.C. Carroll, G. Funke, M.A. Pfaller, M.L. Landry, S.S. Richter, D.W. Warnock, 909-919. doi: <https://doi.org/10.1128/9781555817381.ch51>

Spellerberg, B., Brandt, C. (2015). “*Streptococcus*” in Manual of clinical microbiology, ed. J. H. Jorgensen, K.C. Carroll, G. Funke, M.A. Pfaller, M.L. Landry, S.S. Richter, D.W. Warnock, 226-237. doi: <https://doi.org/10.1128/9781555817381.ch22>

Stomeo, F., Portillo, M. C., Gonzalez, J. M., Laiz, L., Sáiz-Jiménez, C. (2008). Pseudonocardia in white colonizations in two caves with Paleolithic paintings. International Biodeterioration and Biodegradation, 62, 483-486.doi: <https://doi.org/10.1016/j.ibiod.2007.12.011>

Summers Engel, Megan L. Porter, Brian K. Kinkle, Thomas C. Kane, A. (2001). Ecological assessment and geological significance of microbial communities from Cesspool Cave, Virginia. Geomicrobiology Journal, 18, 259-274.doi: <https://doi.org/10.1080/01490450152467787>

Takada, K., Hirasawa, M. (2008). *Streptococcus dentirousetti* sp. nov., isolated from the oral cavities of bats. International journal of systematic and evolutionary microbiology, 58, 160-163.doi: <https://doi.org/10.1099/ijs.0.65204-0>

Tok, E., Olgun, N., Dalfes, H.N. (2021). Profiling Bacterial Diversity in Relation to Different Habitat Types in a Limestone Cave: İnsuyu Cave, Turkey, Geomicrobiology Journal, 38, 776-790.doi: [10.1080/01490451.2021.1949647](https://doi.org/10.1080/01490451.2021.1949647)

Tomova, I., Lazarkevich, I., Tomova, A., Kambourova, M., Vasileva-Tonkova, E. (2013). Diversity and biosynthetic potential of culturable aerobic heterotrophic bacteria isolated from Magura Cave, Bulgaria. International Journal of Speleology, 42. doi: <http://dx.doi.org/10.5038/1827-806X.42.1.8>

Toprak, N. U., Duman, N., Sacak, B., Ozkan, M. C., Sayın, E., Mulazimoglu, L., Soyletir, G. (2021). *Alloprevotella rava* isolated from a mixed infection of an elderly patient with chronic mandibular osteomyelitis mimicking oral squamous cell carcinoma. New Microbes and New Infections, 42. doi: <https://doi.org/10.1016/j.nmni.2021.100880>

Voig, C.C., Caspers, B., Speck, S. (2005). Bats, bacteria, and bat smell: sex-specific diversity of microbes in a sexually selected scent organ. Journal of Mammalogy, 86, 745-749. doi: [https://doi.org/10.1644/1545-1542(2005)086[0745:BBABSS]2.0.CO;2](https://doi.org/10.1644/1545-1542(2005)086%5b0745:BBABSS%5d2.0.CO;2)

Zbinden, R. (2015). “*Aggregatibacter, Capnocytophaga, Eikenella, Kingella*, *Pasteurella*, and Other Fastidious or Rarely Encountered Gram‐Negative Rods” in Manual of clinical microbiology, ed. J.H. Jorgensen, K.C. Carroll, G. Funke, M.A. Pfaller, M.L. Landry, S.S. Richter, D.W. Warnock, 652-666. doi: <https://doi.org/10.1128/9781555817381.ch35>

Zhu, H. Z., Zhang, Z. F., Zhou, N., Jiang, C. Y., Wang, B. J., Cai, L., and Liu, S. J. (2019). Diversity, distribution and co-occurrence patterns of bacterial communities in a karst cave system. Frontiers in microbiology, 10. Doi: <https://doi.org/10.3389/fmicb.2019.01726>

Zou, Y., Lin, X., Xue, W. et al*.* (2021). Characterization and description of *Faecalibacterium butyricigenerans* sp. nov. and *F. longum* sp. nov., isolated from human faeces. Scientific Reports 11.doi: https://doi.org/10.1038/s41598-021-90786-3
